# Routine Optical Clearing of 3D-Cell Cultures: Simplicity Forward

**DOI:** 10.3389/fmolb.2020.00020

**Published:** 2020-02-21

**Authors:** Elina Nürnberg, Mario Vitacolonna, Julia Klicks, Elena von Molitor, Tiziana Cesetti, Florian Keller, Roman Bruch, Torsten Ertongur-Fauth, Katja Riedel, Paul Scholz, Thorsten Lau, Richard Schneider, Julia Meier, Mathias Hafner, Rüdiger Rudolf

**Affiliations:** ^1^Institute of Molecular and Cell Biology, Faculty of Biotechnology, Mannheim University of Applied Sciences, Mannheim, Germany; ^2^Zentralinstitut für Seelische Gesundheit, Department of Translational Brain Research, Medical Faculty Mannheim, Heidelberg University, Mannheim, Germany; ^3^B.R.A.I.N. AG, Zwingenberg, Germany; ^4^TIP Oncology, Merck Healthcare KGaA, Darmstadt, Germany

**Keywords:** optical tissue clearing, spheroid, organoid, glycerol, z-compensation

## Abstract

Three-dimensional cell cultures, such as spheroids and organoids, serve as increasingly important models in fundamental and applied research and start to be used for drug screening purposes. Optical tissue clearing procedures are employed to enhance visualization of fluorescence-stained organs, tissues, and three-dimensional cell cultures. To get a more systematic overview about the effects and applicability of optical tissue clearing on three-dimensional cell cultures, we compared six different clearing/embedding protocols on seven types of spheroid- and chip-based three-dimensional cell cultures of approximately 300 μm in size that were stained with nuclear dyes, immunofluorescence, cell trackers, and cyan fluorescent protein. Subsequent whole mount confocal microscopy and semi-automated image analysis were performed to quantify the effects. Quantitative analysis included fluorescence signal intensity and signal-to-noise ratio as a function of z-depth as well as segmentation and counting of nuclei and immunopositive cells. In general, these analyses revealed five key points, which largely confirmed current knowledge and were quantified in this study. First, there was a massive variability of effects of different clearing protocols on sample transparency and shrinkage as well as on dye quenching. Second, all tested clearing protocols worked more efficiently on samples prepared with one cell type than on co-cultures. Third, z-compensation was imperative to minimize variations in signal-to-noise ratio. Fourth, a combination of sample-inherent cell density, sample shrinkage, uniformity of signal-to-noise ratio, and image resolution had a strong impact on data segmentation, cell counts, and relative numbers of immunofluorescence-positive cells. Finally, considering all mentioned aspects and including a wish for simplicity and speed of protocols – in particular, for screening purposes – clearing with 88% Glycerol appeared to be the most promising option amongst the ones tested.

## Introduction

In the human body, cells grow in clusters, organizing themselves into function-specific tissues and multifunctional organs in all three spatial dimensions. Since *in vitro* monolayer cell cultures do not sufficiently reflect this attribute, they have often been considered to be limited in representing the physiology of organs and tissues (Imamura et al., 2015; Hafner et al., 2017). In two-dimensional (2D) cell culture models, the lack of comprehensive interaction among cells via cell–cell-contacts and between cells with their surrounding extracellular matrix can lead to non-physiological morphology, gene expression, and cellular behavior (Zschenker et al., 2012; Luca et al., 2013). The absence of nutrient and oxygen gradients, as well as restricted migration potential grown on a plastic surface, further contribute to a limited representation of physiology in 2D *in vitro* systems (Duval et al., 2017). During the last decade, there has been a substantial increase in the use of three-dimensional (3D) cell culture models in a large variety of biological fields, ranging from developmental biology (Lancaster et al., 2013) to oncology (Fong et al., 2016; Drost and Clevers, 2018) and drug discovery (Alepee et al., 2014).

Coarsely, 3D-*in vitro* models can be divided into matrix-supported and matrix-free models (Wang et al., 2014). Amongst others, hydrogels, decellularized matrices, porous polymers, and nanofibers might serve as scaffolds in static or dynamic experimental setups can be designed (Das et al., 2015; Carvalho et al., 2017), e.g., in organ-on-a-chip systems (Bauer et al., 2018; Hübner et al., 2018). With respect to matrix-free 3D cultures, spheroids are common due to their ease and reliability of production. Currently, numerous 3D-spheroid models for tissues like skin and its pathological conditions (Chiricozzi et al., 2017; Klicks et al., 2019), tumor (Shroyer, 2016), intestine (Pereira et al., 2016), skeletal muscle (Khodabukus et al., 2018), or brain (Lee et al., 2017) are available.

Despite the widespread usage of 3D-cell culture models, there is much potential for optimization in related analytical downstream processes. The analysis of cell type or marker protein distribution in fixed frozen or paraffin-embedded biological 3D samples typically uses tissue sectioning followed by immunohistological staining, and confocal laser scanning microscopy (CLSM). Due to the time-consuming preparation, potential loss of tissue sections, and the cumbersome reconstruction of spatial 3D-information, such samples are mostly analyzed only partially (Leong, 2004; Berlanga et al., 2011; Marchevsky and Wick, 2015). In addition, this method is destructive and not compatible with high throughput. In samples with homogeneous distribution of cells and effects, this technique might yield representative results (Grootjans et al., 2013; Rohe et al., 2018; Laugisch et al., 2019; Roelofs and De Bari, 2019). However, heterogeneous distribution of different cell types or effects in more complex *in vitro* culture models, such as tumor co- or triple cultures or stem cell-derived organoids, might yield non-representative data upon classical sectioning (Wu and Swartz, 2014; Renner et al., 2017). To circumvent these issues, in toto immunofluorescence of the intact sample followed by whole mount imaging with confocal or light sheet microscopy can be used (Mertz, 2011). However, penetration of light into biological samples is usually limited to around 50–70 μm. Primarily, this is due to light scattering caused by refractive index (RI) mismatches at the interfaces between biological tissue components, such as proteins, water, and lipids (Schmitt and Kumar, 1998). Accordingly, reduction of RI mismatches renders biological tissues optically transparent. Several different optical tissue clearing methods have been developed in the recent years, aiming to improve optical transparency and thus enable fluorescence imaging deep within tissues (Silvestri et al., 2016; Tainaka et al., 2016). Methods based on organic solvents, such as BABB (benzyl alcohol, benzyl benzoate) (Dodt et al., 2007), 3DISCO (3D imaging of solvent-cleared organs) (Erturk et al., 2012), iDISCO (immunolabeling-enabled three-dimensional imaging of solvent-cleared organs) (Renier et al., 2014) and uDISCO (ultimate DISCO) (Pan et al., 2016) are based on combinations of dehydration, delipidation, and homogenization of RI at around 1.55. Although fast, extensive removal of lipids can be detrimental (Ariel, 2017). Furthermore, tissue shrinkage and the use of toxic chemicals, which can affect fluorophore stability, are of disadvantage. Conversely, water-based high-RI solutions provide a more protein-friendly environment and do not affect fluorophore stability as much as organic solvents. Here, RIs of approximately 1.44–1.48 can be achieved by using high concentrations of sugars, such as sucrose or fructose (Tsai et al., 2009; Ke et al., 2013, 2016; Hou et al., 2015). However, these solutions often exhibit a high viscosity. Commercially available ready-to-use tissue clearing products, such as FocusClear (Chiang et al., 2002) and RapiClear (Chen et al., 2019) are convenient but costly. Furthermore, aqueous high-RI solutions often display limited clearing efficiency in larger tissues, making them more adequate for smaller structures, such as spheroids, organoids, or tissue slices (Ariel, 2017). In contrast to these methods, hyperhydrating clearing protocols reduce the RI of tissue to ∼ 1.38–1.48 and often employ removal of light scattering molecules. For example, the Sca*l*e-method (Hama et al., 2011) uses a combination of delipidation, maintenance of the aqueous environment, and urea-mediated tissue hydration, which partially denatures and hydrates high-RI proteins (Richardson and Lichtman, 2015). Implementation of amino alcohol-mediated tissue decolorization shortened clearing times, leading to methods such as Sca*l*eCUBIC (Sca*l*e clear, unobstructed brain imaging cocktails and computational analysis) (Susaki et al., 2014) and Sca*l*eS (sorbitol-based Sca*l*e) (Hama et al., 2015). Detergent-free hyperhydrating clearing methods (Kuwajima et al., 2013) make use of gradients of aqueous formamide solutions (Clear^T^), or formamide and polyethylene glycol (Clear^T2^). To prevent excessive protein-loss by strong detergents or masking of epitopes by protein-denaturation, tissue transformation methods, such as SWITCH (system-wide control of interaction time and kinetics of chemical) (Murray et al., 2015), CLARITY (clear lipid-exchanged acrylamide-hybridized rigid imaging/immunostaining/*in situ* hybridization-compatible tissue-hYdrogel) (Chung et al., 2013), and its derivates (Yang et al., 2014) include embedding of tissue into a gel-like structure. Despite their great clearing performance, clearing time can vary and technical implementation is complex.

While most available clearing methods were established and optimized in whole organs and tissues, they have recently also been implemented into the analytical pipelines with 3D-cell culture models (Chen et al., 2013, 2016, 2019; Wenzel et al., 2014; Dingle et al., 2015; Kabadi et al., 2015; Masson et al., 2015; Grist et al., 2016; Schmitz et al., 2017; Smyrek and Stelzer, 2017; Costa et al., 2018a; Desmaison et al., 2018). As for organs (Xu et al., 2019), alterations in optical clearing efficiency between different cell types/lines were also observed in 3D-*in vitro* systems (Dingle et al., 2015; Boutin et al., 2018), but systematic studies in that respect have been missing. Therefore, our aim was to compare different optical clearing protocols on a set of spheroids and complex 3D-co-culture systems with respect to maintenance of sample integrity, clearing efficiency, and downstream automated analysis.

## Materials and Methods

A list of all cell lines and reagents used in this study can be found in Table 1 and Supplementary Table S1, respectively.

**TABLE 1:** Cell lines used in the study.

	**Cell line**	**Cell type**	**Labeling**	**Source**
**Mono-cultures**	HaCaT	Keratinocyte	–	B.R.A.I.N. AG
	B7_033#1NPC1	iPSC-derived neural precursor	–	Hector Institute for Translational Brain Research, Mannheim
	CCD-1137SK	Fibroblast	–	ATCC
	HT29	Colorectal adenocarcinoma	–	ATCC
	HTC-8	Human taste bud	–	B.R.A.I.N. AG

**Dynarray co-culture**	MDA-MB231	Triple-negative breast cancer	ECFP	ATCC

	CCD-1137SK	Fibroblast	CellTracker Red	ATCC
**Melanoma tri-culture**	HaCaT	Keratinocyte	CellTracker Red	B.R.A.I.N. AG
	SK-MEL-28	Melanoma	CellTracker Green	ATCC
	CCD-1137SK	Fibroblast		ATCC

*Cell line, cell type, labeling and source are indicated.*

### Cell Culture

To investigate the influence of optical tissue clearing protocols on cells of different origin and 3D-cultivation methods, a total of seven tumorous and non-malignant cell lines was chosen for the generation of mono- and tri-culture spheroids, and a microcavity array-based co-culture model: human keratinocyte cell line HaCaT, human induced pluripotent stem cell-derived neural precursor cells B7_033#1NPC1, human fibroblast cell line CCD-1137SK, colorectal adenocarcinoma-associated HT29 cells, human tongue cell line HTC-8, melanoma cell line SK-MEL-28, and the stably transfected, breast cancer-associated cell line MDA-MB-231-ECFP. All cell lines were repeatedly authenticated by phenotypic analysis and regularly tested for mycoplasma.

### Spheroid Generation

For all mono- and tri-culture spheroids used in this study, the appropriate amounts of cells were seeded onto ultra-low attachment (ULA) 96-well U-bottom plates (Corning) in their corresponding medium and centrifuged at 300 rpm for 5 min. All cells were maintained in a humidified incubator at 37°C and 5% CO_2_ and, with the exception of a melanoma tri-culture model, kept in culture until reaching a diameter of ∼ 300 μm.

#### HaCaT Spheroids

The human keratinocyte cell line HaCaT (B.R.A.I.N. AG) was cultured in Dulbecco’s Modified Eagle Medium (DMEM, Capricorn) supplemented with 1% penicillin/streptomycin (Pen/Strep, Sigma-Aldrich) and 10% fetal bovine serum (FBS, Capricorn). For spheroid generation, cells were detached using Trypsin/EDTA (Sigma-Aldrich) and seeded onto 96-well ULA plates at a concentration of 9 × 10^3^ cells per well. A diameter of ∼300 μm was reached after four days of cultivation.

#### B7_033#1NPC1 Spheroids

The human iPSC-derived B7_033#1 NPC1 line was kindly provided by S. Horschitz (Hector Institute for Translational Brain Research, Mannheim, Germany) and cultured in DMEM/Ham’s F12 + GlutaMAX supplemented with 1% N2 and 2% B27 supplements, 1% GlutaMAX, 1% Minimum Essential Medium Non-essential Amino Acids (MEM-NEAA, all Invitrogen), 1% Pen/Strep and 20 ng/mL FGF-2_Type 147 (Cell Guidance Systems). For spheroid generation, cells were detached using TrypLE (Invitrogen) and seeded onto 96-well ULA plates at a concentration of 1 × 10^4^ cells per well. A diameter of ∼ 300 μm was reached after three days of cultivation.

#### CCD-1137SK Spheroids

CCD-1137SK fibroblast cells derived from human foreskin (ATCC) were cultured in Iscove’s Modified Dulbecco’s Medium (IMDM, Capricorn) supplemented with 10% FBS and 1% Pen/Strep. For spheroid generation, cells were detached using Trypsin/EDTA and seeded onto 96-well ULA plates at a concentration of 1 × 10^4^ cells per well. An average diameter of 300 μm was reached after four days of cultivation.

#### HT29 Spheroids

HT-29 colon cancer cells (ATCC) were cultured in McCoy’s 5A medium (Capricorn) supplemented with 10% FBS and 1% Pen/Strep. For spheroid generation, cells were detached using Trypsin/EDTA and seeded onto 96-well ULA plates at a concentration of 1 × 10^3^ cells per well. A diameter of ∼ 300 μm was reached after three days of cultivation.

#### HTC-8 Spheroids

Human tongue cell line HTC-8 (B.R.A.I.N. AG) was cultured in HTC-medium according to Hochheimer et al. (2014). For spheroid generation, cells were detached using Trypsin/EDTA and a concentration of 6 × 10^3^ cells per well were seeded onto 96-well U-bottom plates. An average diameter of 300 μm was reached after five days of cultivation.

#### Melanoma Tri-Culture Spheroids

Melanoma tri-cultures were generated according to Klicks et al. (2019). Briefly, 1 × 10^4^ cells of CCD-1137SK cells were seeded, followed by simultaneous addition of HaCaT (1 × 10^4^ cells/well) and SK-MEL-28 (ATCC) (2.5 × 10^3^ cells/well) after three days. For discrimination of individual cell types, HaCaT and SK-MEL-28 cells were labeled with CellTracker Red CMPTX (10 μM) and CellTracker Green CMFDA (10 μM) dye (both Life Technologies), respectively according to manufacturer instructions for 30 min. Tri-culture spheroids were kept in culture for another two days to reach an average diameter of 300 μm.

#### Dynarray Co-culture Model

The human breast cancer cell line MDA-MB231-ECFP was maintained in DMEM supplemented with 10% FBS, 1% MEM-NEAA and 1% Pen/Strep. For co-culture experiments in Dynarrays (300MICRONS), MDA-MB-231-ECFP and CCD-1137SK cells were used. MDA-MB-231 (ATCC) were stably transfected with ECFP-C1 plasmid (Addgene) using Nucleofector^TM^ II (Lonza) following the manufacturer’s protocol. Briefly, ECPF plasmid was linearized by incubating 10 μg/μL DNA with 10% μL *Ase*I buffer 3.1 (New England Biolabs) and 5% *Ase*I enzyme (New England Biolabs) for 30 min at 37°C under gentle shaking with 300 rpm. To keep the construct in linear shape, DNA was incubated with alkaline phosphatase (Invitrogen) for 30 min at 37°C. 1 × 10^6^ MDA-MB-231 cells were transfected using Nucleofector II and 2 μg linearized ECFP. CCD-1137SK were labeled with CellTracker Red CMPTX according to manufacturer instructions at a concentration of 10 μM in serum-free medium for 30 min. Prior to cell seeding, Dynarrays were treated with an isopropyl alcohol cascade (100%, 70%, 50%, 30% of alcohol in purified water for 30 s each). After washing twice with purified water, chips were coated for 1 h at room temperature with 30 μg/ml rat tail collagen 1 (Roche) in 0.2% acetic acid and washed once with phosphate-buffered saline (PBS). 2 × 10^6^ MDA-MB231-ECFP and 2 × 10^6^ CellTracker labeled CCD-1137SK were mixed in 100 μl media and seeded onto Dynarray chips. Following a 3 h incubation at 37°C in a CO_2_ incubator, 10 ml DMEM were added and changed every other day for nine days.

### Fixation and Immunofluorescence

Spheroids were transferred into 200 μl PCR-tubes, washed 2 x with PBS and fixed with 4% paraformaldehyde (PFA, Carl Roth) for 1 h at 37°C, followed by washing twice with PBS containing 1% FBS, for 5 min each. Then, spheroids were quenched with 0.5 M glycine (Carl Roth) in PBS for 1 h at 37°C with gentle shaking. With the exception of Sca*l*eS clearing protocol, all other optical clearing methods contained the following steps for immunofluorescence: after fixation and PFA-quenching, samples were incubated in penetration buffer for 30 min, containing 0.2% Triton X-100, 0.3 M glycine, and 20% DMSO (all Carl Roth) in PBS to improve penetration of antibodies and nuclear dyes. Spheroids were then washed twice with PBS/1% FBS and incubated in blocking buffer (0.2% Triton X-100, 1% bovine albumin serum (BSA, Carl Roth), 10% DMSO in PBS) for 2 h at 37°C with gentle shaking. After blocking, samples were incubated with primary antibody overnight (ON) at 37°C with gentle shaking. Primary anti-KI67 antibody (Merck, rabbit polyclonal antibody) was diluted 1:300 in antibody buffer (0.2% Tween 20, 10 μg/ml heparin (both Sigma-Aldrich), 1% BSA, 5% DMSO in PBS). Samples were then washed 5 times for 5 min each in washing buffer (0.2% Tween 20, 10 μg/mL heparin, 1% BSA) and stained with secondary antibody and nuclear dyes ON at 37°C with gentle shaking. Corresponding secondary antibody and nuclear dyes were diluted in antibody buffer with the following concentrations: donkey anti-rabbit AlexaFluor488, 1:800; donkey anti-rabbit AlexaFluor647, 1:800; DRAQ5, 1:1000 (all Invitrogen); DAPI, 1:500 (Sigma-Aldrich). Samples were washed subsequently 5 times for 5 min in washing buffer with gentle shaking and then embedded or cleared according to the following protocols, protected from light to prevent bleaching of fluorophores.

### Optical Clearing

Embedding in PBS or Mowiol (Sigma-Aldrich) and optical clearing with Glycerol: after immunofluorescence and 24 h prior to imaging, samples were mounted in 18 well μ-slides (ibidi) in either PBS or Mowiol. Glycerol-based RI matching was performed according to Williams et al. (2019) by immersion of stained spheroids in an aqueous solution of 88% Glycerol (RI 1.459) ON at RT with gentle shaking, followed by mounting on 18 well μ-slides in the same solution. All procedures were executed with minimal exposure to light. After mounting, spheroids were always kept in the microscope room for several hours to allow for temperature adjustment prior to microscopy.

#### Clear^T2^

This was performed according to Dingle et al. (2015). Briefly, fixed spheroids were incubated for 10 min at RT in a solution of 25% formamide/10% polyethylene glycol (PEG) (both Carl-Roth), followed by a 5 min incubation in a 50% formamide/20% PEG solution. Finally, samples were immersed in fresh 50% formamide/20% PEG and incubated for 60 min at RT and subsequently mounted. All steps were carried out under gentle movement.

#### CytoVista

After immunofluorescence staining, remaining liquid was carefully removed from samples, followed by ON immersion at RT in 30 μL of CytoVista Tissue Clearing Reagent (Invitrogen). Then, spheroids were mounted in ibidi 18 well μ-slides in CytoVista reagent.

#### Sca*l*eS

An adapted version of the original Sca*l*eS tissue clearing protocol (Hama et al., 2015) for indirect immunofluorescence was used. After fixation, spheroids were immersed ON in S0 solution (20% (w/v) D-sorbitol (Sigma-Aldrich), 5% (w/v) Glycerol (Carl-Roth), 3% (v/v) DMSO in PBS, pH 7.2) to avoid sample floating in subsequent steps. Permeabilization involved adaptation in S0, immersion in A2 (10% (w/v) Glycerol, 4 M urea (Sigma-Aldrich), 0.1% (w/v) Triton X-100 in ddH_2_O, pH 7.7) for 24 h at 37°C, followed by a 24 h incubation in B4(0) (8 M urea in ddH_2_O, pH 8.4) at 37°C and a final incubation in A2 ON at 37°C. Then, samples were washed for 6 h in PBS at RT and blocked in Sca*l*eS blocking solution (2.5% (w/v) BSA, 0.05% (w/v) Tween 20, 0.1% (w/v) Triton X-100 in PBS, pH 7.4) for 24 h at 37°C. Then, samples were incubated with primary antibody for 24 h at 37°C, washed two times for 2 h each in fresh AbSca*l*e (0.33 M urea, 0.1% (w/v) Triton X-100 in PBS, pH 7.4) at RT and then incubated ON at 37°C in secondary antibody solution containing nuclear dyes. Next, samples were washed in fresh AbSca*l*e for 6 h at RT, followed by reblocking twice for 2 h each in Sca*l*eS blocking solution. After refixation in 4% PFA for 1 h at RT, spheroids were washed in PBS ON at 4°C. For RI matching, samples were incubated in Sca*l*eS4 solution (40% (w/v) D-sorbitol, 10% (w/v) Glycerol, 4 M urea, 15% DMSO in ddH_2_O, pH 8.1) ON at RT and mounted in 18 well μ-slides. All steps were carried out under gentle movement to ensure proper immersion of samples. After mounting, spheroids were kept in the microscope room for several hours to allow temperature adjustment.

### Image Acquisition and Analysis

**FIGURE 1:**
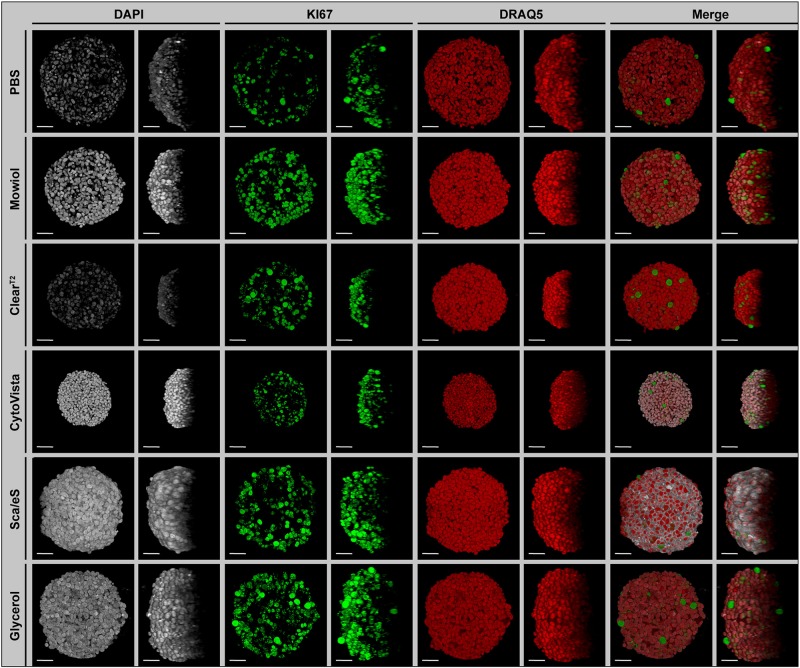
Preservation of fluorescence signal intensity, sample volume and optical transparency is strongly dependent on optical clearing protocol. Upon growth to a diameter of approximately 300 μm, spheroids made of HaCaT keratinocytes were fixed, stained with anti-KI67 (green) and the nuclear dyes, DAPI (gray) and DRAQ5 (red), followed by optical tissue clearing or embedding as indicated and subsequent confocal whole mount microscopy. Images show representative top (**Left panels** for each staining) and orthogonal (**Right panels** for each staining) 3D volume projections of single and merged channels after corresponding clearing method. Scale bars, 50 μm.

**FIGURE 2:**
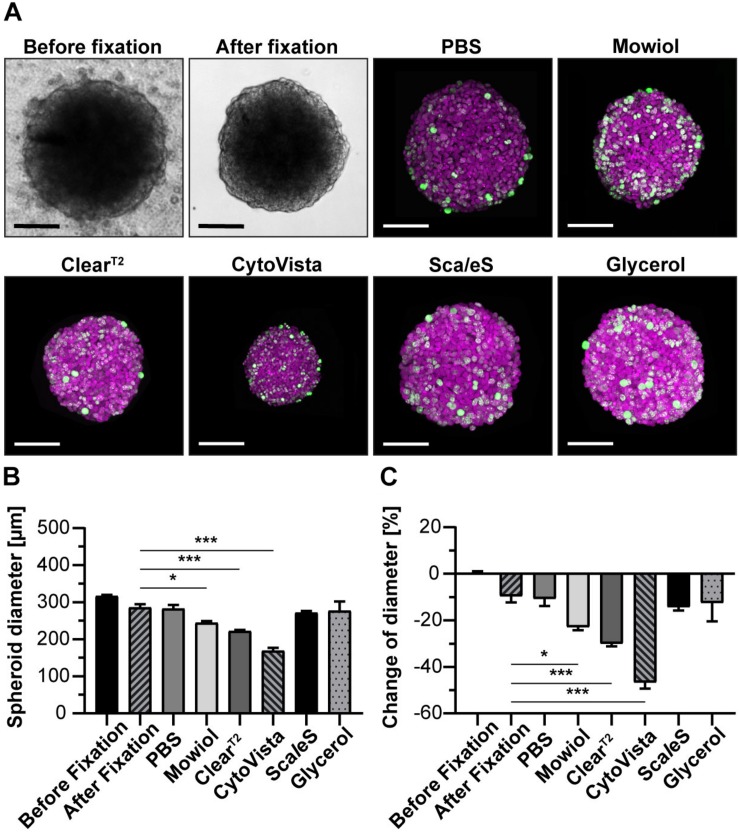
Aqueous clearing methods and detergent-containing hyperhydration prevent massive post-fixation volume changes. Upon growth to a diameter of approximately 300 μm, spheroids made of HaCaT keratinocytes were fixed, stained with anti-KI67 and DRAQ5, followed by optical tissue clearing or embedding as indicated. Spheroid diameters were determined from brightfield images before and after fixation and from confocal microscopy stacks after staining. **(A)** Representative brightfield images of spheroids before and after fixation as well as maximum projections of confocal image stacks upon staining and clearing/embedding as indicated. In the confocal panels, DRAQ5 and KI67 fluorescence signals are shown in magenta and green, respectively. Scale bars, 100 μm. Quantitative analysis of average spheroid diameter **(B)** and change of average spheroid diameter relative to pre-fixation state **(C)**. Graphs depict mean + standard deviation (SD); *n* ≥ 9; *p ≤ 0.05, ***p ≤ 0.001.

**FIGURE 3:**
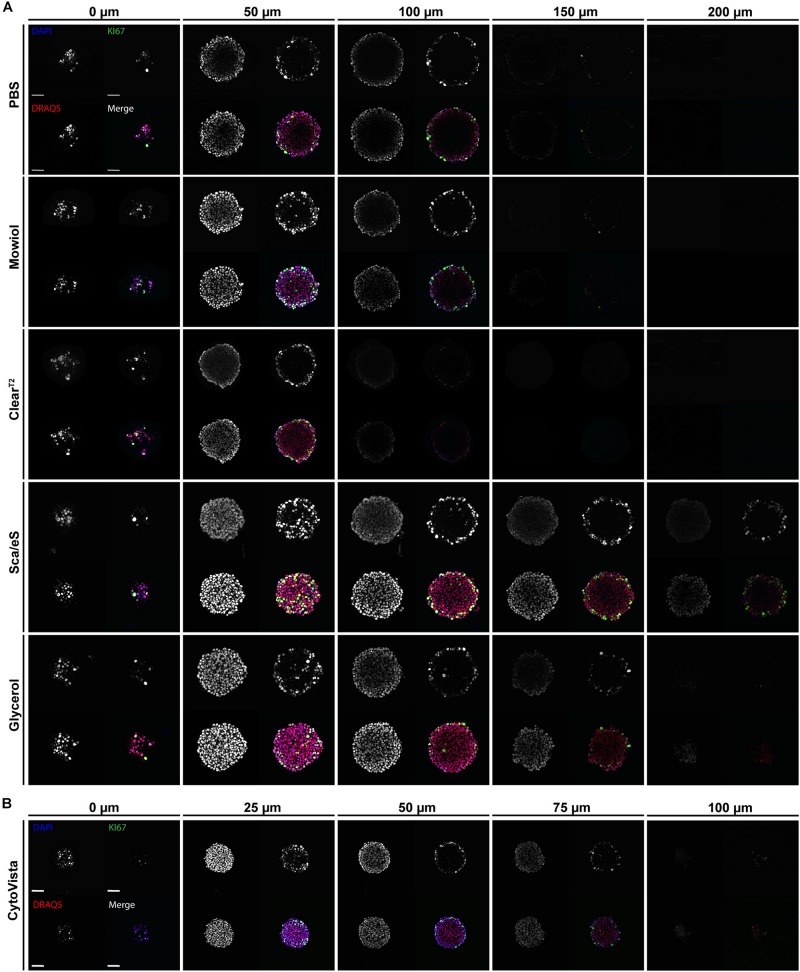
High-refractive index aqueous solutions or detergent-containing hyperhydration improve light penetration into spheroids. Upon growth to a diameter of approximately 300 μm, spheroids made of HaCaT keratinocytes were fixed, stained with anti-KI67, DAPI, and DRAQ5, followed by optical tissue clearing or embedding as indicated and subsequent confocal whole mount microscopy. **(A,B)** Depicted are single optical sections of spheroids at discrete z-depths with fixed intervals of 50 μm **(A)** or 25 μm **(B)**. Each panel shows DAPI, DRAQ5, and KI67 fluorescence signals, as well as a merge. In merged panels DAPI is shown in blue, DRAQ5 in red, and KI67 in green. Colocalization of DAPI and DRAQ5 appears in magenta, additional colocalization with KI67 in green to white hues. Scale bars 50 μm.

**FIGURE 4:**
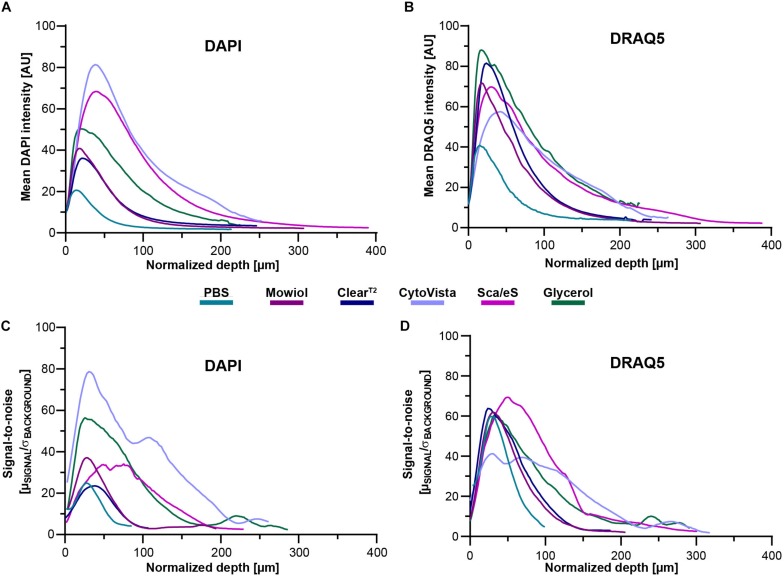
Optical transparency, preservation of fluorescence signals and depth-dependent SNR are dependent on clearing method and cell line. Mono-culture spheroids of HaCaT cells were grown to approximately 300 μm diameter and then fixed. Upon staining of proliferating cells (anti-KI67) and nuclei (DAPI and DRAQ5), spheroids were embedded/cleared as indicated and then imaged in toto using confocal microscopy. Analysis of depth-dependent signal intensity of DAPI and DRAQ5 was performed by selecting one circular region of interest (ROI) per sample through the central region of the spheroid followed by mean intensity measurement throughout the entire stack depth. SNR values for DAPI and DRAQ5 were determined by measurement of mean intensity and standard deviation of background and nuclear signal via semi-automated thresholding. Then, SNR was calculated as the ratio of mean signal intensity in identified nuclear regions to the average standard deviation of background intensity (μ_SIGNAL_/σ_BACKGROUND_). To account for volume-changing effects of individual clearing methods, all depth values were normalized by the method-dependent degree of swelling or shrinkage. **(A,B)** Graphs show mean intensities of DAPI and DRAQ5 as a function of normalized z-depth HaCaT spheroids. **(C,D)** Graphs show mean SNR values for staining with DAPI and DRAQ5 as a function of normalized z-depth for HaCaT spheroids. All mean values were calculated from n ≥ 7 spheroids per condition.

**FIGURE 5:**
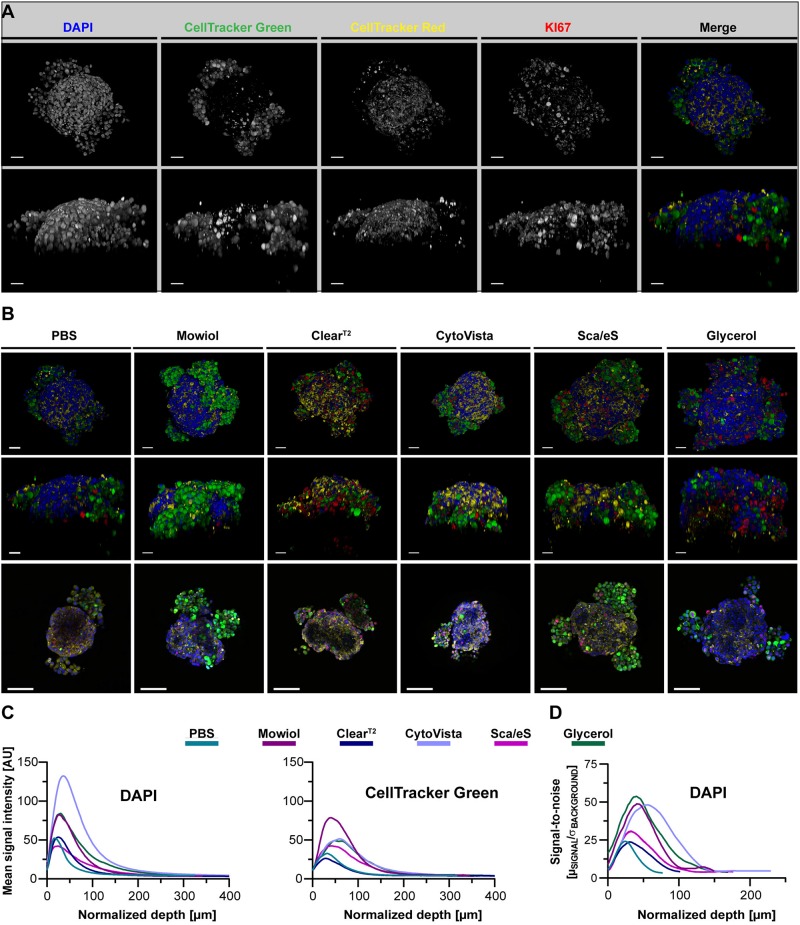
Optical clearing efficiency is affected by complexity of a spheroid-based tri-culture model. Melanoma tri-culture spheroids were generated starting with formation of a core spheroid of CCD-1337SK fibroblasts, followed after 72 h by simultaneous addition of HaCaT keratinocytes labeled with CellTracker Red and SK-MEL-28 melanoma cells marked with CellTracker Green. After another two days of cultivation, spheroids were fixed, stained with anti-KI67 and DAPI, and then embedded or cleared as indicated. Confocal 3D-imaging of whole spheroid cultures was performed. **(A)** Representative top and orthogonal 3D-volume projections of fluorescence signals (indicated) of a PBS-embedded spheroid are shown in upper and lower panels, respectively. In the merge, DAPI appears in blue, CellTracker Green in green, CellTracker Red in yellow, and KI67 in red. Scale bars, 50 μm. **(B)** Depicted are representative images of tri-culture spheroids after different types of embedding/clearing as indicated. Top and side view maximum projections are shown in upper and middle panels. Lower panels show single optical sections at 75 μm of imaging depth. Scale bars, 50 μm in upper and middle row, 100 μm in lower row. Quantitative analysis of mean signal intensity of DAPI and CellTracker Green **(C)** and SNR of DAPI signals **(D)** as a function of normalized depth. Mean of *n* = 10 spheroids per condition.

**FIGURE 6:**
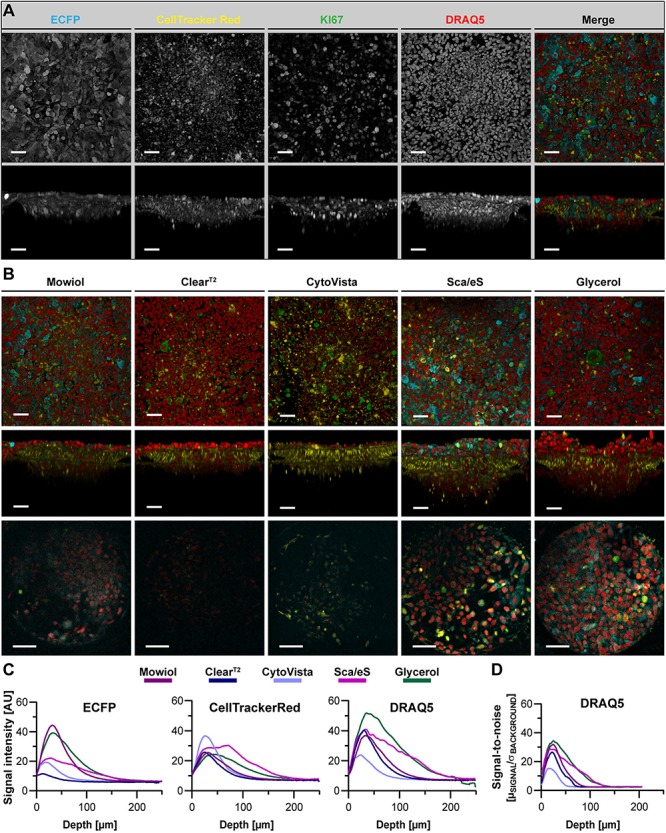
ECFP fluorescence is maintained by Glycerol-RI matching in a chip-based co-culture model of breast cancer cells and fibroblasts. ECFP-expressing MDA-MB-231 breast cancer cells were co-seeded with CellTracker Red labeled CCD-1337SK fibroblasts into Dynarray chips with 300 μm wide cavities. After 9 days of cultivation, chips were fixed, stained with anti-KI67 and DRAQ5, and then embedded or cleared as indicated. **(A)** Representative top and orthogonal 3D-volume projections of fluorescence signals (indicated) of Mowiol-embedded chip cavities are shown in upper and lower panels, respectively. In the merge, ECFP appears in cyan, CellTracker Red in yellow, KI67 in green, and DRAQ5 in red. Scale bars, 50 μm. **(B)** Depicted are representative images of chip cavities after different types of embedding/clearing as indicated. Top and side view maximum projections are shown in upper and middle panels. Lower panels show single optical sections at 75 μm of imaging depth. Scale bars, 50 μm in upper and middle row, 100 μm in lower row. **(C,D)** Quantitative analysis of mean signal intensity of ECFP, CellTracker Red, and DRAQ5 **(C)** and SNR of DRAQ5 signals **(D)** as a function of depth. Mean of *n* = 10 cavities per condition.

**FIGURE 7:**
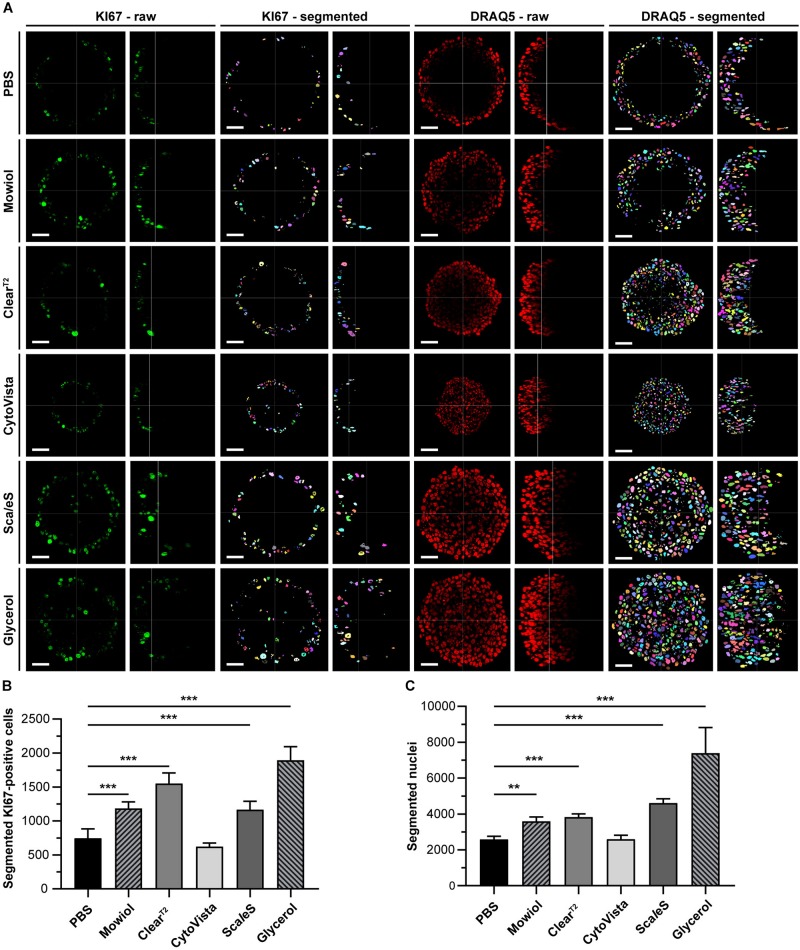
Optical clearing of HaCaT spheroids with Glycerol improves detection and quantification of cell nuclei. Upon growth to a diameter of approximately 300 μm, spheroids made of HaCaT keratinocytes were fixed, stained with anti-KI67 and DRAQ5, followed by optical tissue clearing or embedding as indicated and subsequent confocal whole mount microscopy. Semi-automated image segmentation was performed to detect and count KI67^+^ and DRAQ5^+^ nuclei. **(A)** Depicted are raw single optical sections and orthogonal views from central regions for both markers (KI67 – raw; DRAQ5 – raw) and corresponding segmented images (KI67 – segmented; DRAQ5 – segmented). In raw images, fluorescence signals of KI67 and DRAQ5 are shown in green and red, respectively. Different colors of segmented nuclei were used for better visual discrimination. Scale bars, 50 μm. B-C: Quantitative analysis of KI67^+^ **(B)** and Draq5^+^ nuclei **(C)** as a function of clearing/embedding protocol. Data show mean + SD; *n* ≥ 9; **p ≤ 0.01; ***p ≤ 0.001.

**FIGURE 8:**
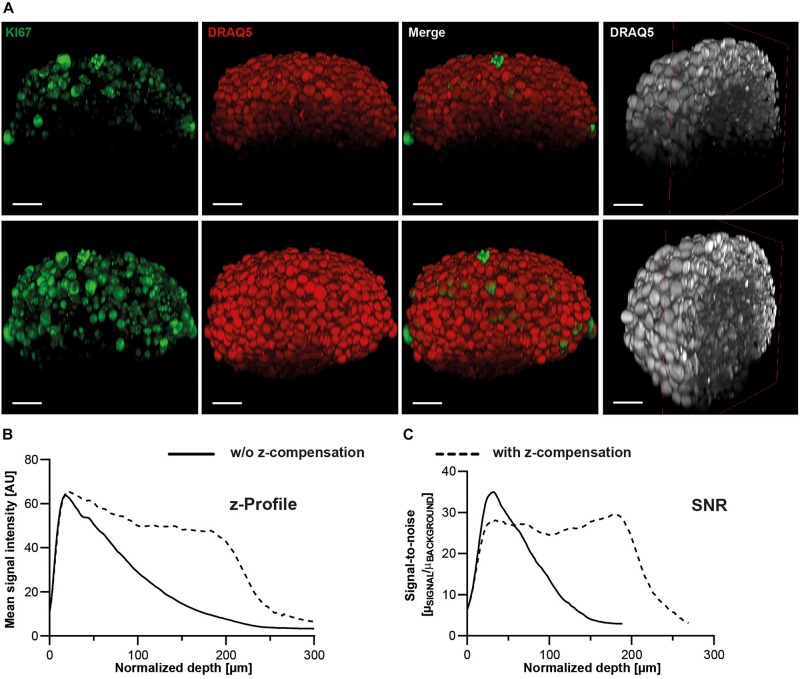
Z-profiles of signal intensity and SNR remain more stable upon z-compensation. Upon growth to a diameter of approximately 300 μm, spheroids made of HaCaT keratinocytes were fixed, stained with anti-KI67 and DRAQ5, followed by optical tissue clearing with Glycerol and subsequent confocal whole mount microscopy. **(A)** Depicted are orthogonal maximum projections (left three columns) and a clipped projection to visualize the spheroid core (right column) of the same spheroid imaged in the absence (upper panels) or presence of z-compensation (lower panels). In the left panels, fluorescence signals of KI67 and DRAQ5 are shown in green and red, respectively. The right panels show only DRAQ5 signals in gray. Scale bars, 50 μm. Graphical representations of z-profiles for DRAQ5 channel signal intensity **(B)** and SNR **(C)** in a z-extended column through the central region of the spheroid.

**FIGURE 9:**
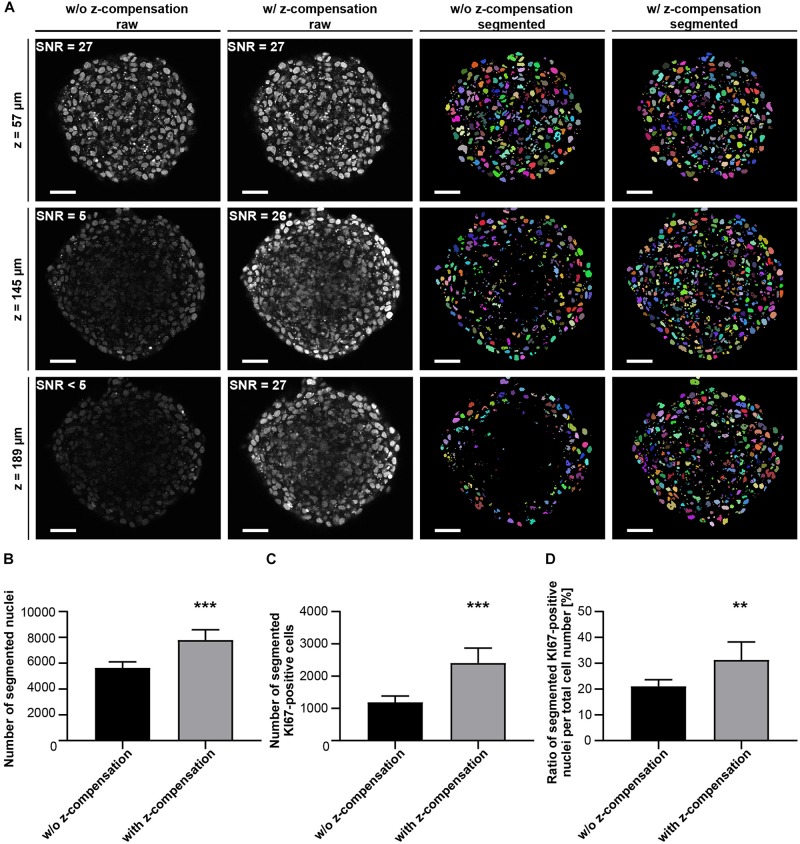
Z-compensation improves semi-automated segmentation of nuclei. Upon growth to a diameter of approximately 300 μm, spheroids made of HaCaT keratinocytes were fixed, stained with anti-KI67 and DRAQ5, followed by optical tissue clearing with Glycerol and subsequent confocal whole mount microscopy. **(A)** Depicted are single raw confocal images (left two columns) and corresponding segmented nuclei (right two columns) at different z-depths showing DRAQ5 signals in gray and segmented nuclei in different colors. The corresponding image stacks were taken in the absence or presence of z-compensation, as indicated. SNR values measured in the shown raw images are given in their upper left angles. Scale bars, 50 μm. Quantitative analysis of Draq5^+^ nuclei **(B)**, KI67^+^ nuclei **(C)**, and ratio of KI67^+^/DRAQ5^+^ nuclei **(D)** in the absence or presence of z-compensation. Data show mean + SD; *n* ≥ 9; **p ≤ 0.01; ***p ≤ 0.001.

For microscopic imaging, an inverted Leica TCS SP8 confocal microscope (Leica Microsystems CMS, Mannheim) equipped with HC PL APO 20 x/0.75 IMM CORR objective, 405 nm, 488 nm, 561 nm, and 633 nm lasers and Leica Application Suite X software was used. All image stacks were acquired with comparable settings, using Immersion Type F (Leica Microsystems, RI 1.52), at a resolution of 1024 × 1024 pixels, z-step size of 1.5 μm, a laser intensity of 1–1.5% and a gain between 600 and 750 V, in order to avoid overexposure of pixels. For comparability of tested clearing methods, image stacks for Figures 1–7 and Supplementary Figures S1–S6 were acquired without z-compensation. Data for Figures 8, 9 were acquired with z-compensation. For brightfield microscopy, an Axiovert 25 (Carl Zeiss Microscopy GmbH) was used.

Measurement of spheroid diameter and depth-dependent z-axis profile: the average size of each spheroid before and after fixation was determined by measuring the sample diameter twice in a perpendicular angle using the line tool of FIJI (Schindelin et al., 2012) on maximum z-projections made from image stacks acquired by confocal microscopy. For measuring the depth-dependent signal intensity, a circular region of interest (ROI) with a diameter of 100 μm was placed in the central region of each spheroid. The ROI area was duplicated from the original image stack and the mean signal intensity determined for each single optical section by using FIJIs function to plot a z-axis profile.

#### Signal-to-Noise Ratio

For the depth-dependent SNR, ROI-stacks for the measurement of signal intensity were used to determine intensity values of background and nuclear signal. Briefly, a median filter (radius 1) was applied to image stacks from a central region of each spheroid and a threshold range was manually set in upper regions of the sample, to cover areas defined as background, and converted into binary masks. ROIs generated from binary masks were used to automatically measure mean background intensity and standard deviation from the initial image stack. For determination of signal intensity of nuclear dyes, an automated thresholding mechanism was applied independently to each optical section and images were converted into binary masks, which were used to measure signal intensities from initial stacks. SNR was calculated for each optical section and defined as:

$$\mathrm{SNR}=\frac{\mu_{\mathrm{SIGNAL}}}{\sigma_{\mathrm{BACKGROUND}}}$$

where μ depicts mean intensity of signal and σ the standard deviation of background intensity. In order to measure excitation wavelength-dependent differences in SNR, DAPI and DRAQ5 were analyzed.

#### 3D-Segmentation of Nuclei

3D confocal data were pre-processed using Fiji. This included image cropping, splitting of multichannel datasets into single channel stacks to segment cell nuclei and antibody staining separately, background correction, conversion into 8-bit format, and export as tiff files. Segmentation and quantification of stacks was performed with a previously published algorithm (Schmitz et al., 2017) which employs 3D-seeded watershed for segmentation. Parameter values were adjusted using the build-in graphical user interface. The median filter range was set to 3 pixels, local threshold range to 10 pixels, and hole filling range to 1 pixel. For seed detection, Laplace of Gaussian (LoG) with a seed range between 9 and 25 pixels was chosen. The other parameters were applied as default. Post-segmented data were exported as different 3D stacks in tiff format and a XLSX file containing quantitative results.

#### Statistical Analysis

Statistical analysis employed the program GraphPad Prism 8. Data were tested for normal distribution by Kolmogorov-Smirnov test. For statistical analysis of spheroid size measurements, non-parametric Kruskal-Wallis with Dunn’s multiple comparisons test was performed. Analysis of segmentation results was done by one-way ANOVA with Tukey’s multiple comparisons test. Significance level α was set to 0.05 with 95% confidence interval and p-values were adjusted to account for multiple comparisons.

## Results

To analyze the impact of optical clearing methods on different 3D-*in vitro* systems of varying complexity, mono-culture spheroids, co-culture spheroids, and chip-based 3D-co-cultures were compared. Mono-culture spheroids were prepared with an average diameter of 300 μm were made of cell lines derived from human keratinocytes (HaCaT), fibroblasts (CCD-1137SK), colon cancer-associated cells (HT29), human tongue cells (HTC-8) and human induced pluripotent stem cell (hiPSC)-derived neural precursor cells (B7_033#1NPC1). The more complex cell cultures consisted of a tri-culture model of skin cancer and a microarray-based co-culture model of human fibroblasts and breast cancer cells (MDA-MB231). For convenience of implementation into the lab routine, we chose to analyze clearing efficiencies of simple clearing protocols Clear^T2^, CytoVista, a modified version of Sca*l*eS, and immersion in 88% Glycerol.

### Spheroid Clearing Affects Fluorescence, Transparency, and Sample Volume

First, the optical clearing protocols were applied to all mono-culture spheroids upon triple-labeling with DAPI, DRAQ5, and anti-KI67 antibody. Alternative to optical clearing with Clear^T2^, CytoVista, Sca*l*eS and Glycerol, spheroids were embedded in PBS or Mowiol. As shown in Figure 1 for HaCaT spheroids (for an overview of the other cell types, see Supplementary Figure S1), the brightness of DAPI signals varied strongly between the tested clearing methods. In particular, it was decreased in PBS and Clear^T2^ samples, whereas Mowiol, CytoVista, Sca*l*eS, and Glycerol retained bright DAPI signals. Fluorescence intensities of KI67 and DRAQ5 signals did apparently not differ strongly (Figure 1). Orthogonal volume projections (Figure 1, right panels for each dye) revealed a superior improvement of imaging depth upon clearing with Sca*l*eS and Glycerol. Small spheroid volumes were seen with Clear^T2^ and CytoVista (Figure 1). In the following, the observed changes were analyzed quantitatively.

### Volume Changes of Spheroids Are Minimal With Sca*l*eS and Glycerol

To determine the effects of clearing procedures on sample volume, spheroid diameters were determined from confocal image stacks. Additionally, original sample size and potential post-fixation changes of spheroid diameters were determined from bright field images taken before and after fixation. Figure 2 demonstrates that Mowiol, Clear^T2^, and CytoVista caused significant reductions of HaCaT spheroid diameters, with the strongest effect for CytoVista (44.8 ± 0.7% loss). Conversely, Sca*l*eS and Glycerol did affect spheroid sizes only to a minor extent and compared to the diameters right after fixation, these were unaltered. Similar results were obtained for all other cell lines and tri-culture spheroids (Supplementary Figures S2, S3), although shrinkage was less pronounced for HT29 and HTC-8 cells and B7_033#1NPC1 cells showed a slight volume increase.

### Sca*l*eS and Glycerol Improve Light Penetration Into Spheroids

To analyze light penetration into spheroids, single equidistant optical sections were prepared from top to bottom of the samples. Supplementary Table S2 summarizes the percentage decrease in light penetration of DAPI and Draq5 stains. In PBS, Mowiol, and Clear^T2^ samples, central regions of the spheroids already darkened at 50 μm of depth and signals were almost completely invisible at ∼ 150 μm of depth (Figure 3A). Conversely, Sca*l*eS or Glycerol clearing preserved signal intensities at 100 μm of depth and central spheroid regions remained visible beyond 150 μm for DRAQ5 and KI67 (Figure 3A). Ring-like localization of KI67 signals at 100 μm depth and below was as expected, because cell proliferation in HaCaT spheroids is limited to the spheroid rim. Due to the massive shrinkage of CytoVista-cleared spheroids, optical sections were here analyzed at 25 μm intervals (Figure 3B). Normalized to spheroid size, light penetration was comparably good for Sca*l*eS, Glycerol, and CytoVista samples.

### Signal Intensity in Depth Depends on Clearing Method, Cell Line, and Fluorophore

To quantify the decay of DAPI and DRAQ5 signal intensities as a function of depth, mean intensities at each optical section were measured in circular ROIs placed in the central region of each spheroid. To compensate for clearing-induced volume changes of samples, depth values were normalized to pre-fixation diameters multiplied with the percentage change of swelling or shrinkage (normalized depth). Mean data were plotted for HaCaT spheroids in Figures 4A,B, and for all other cell lines in Supplementary Figure S4. This showed that different clearing protocols can be better suited to either DAPI or DRAQ5, or to a specific cell line. In all spheroids, maximal brightness values were seen in the upper parts of spheroids, followed by exponential decays. As indicated by the 50% and 90%-signal decay depths, Glycerol or Sca*l*eS were superior for most of the samples (Supplementary Table S3). CytoVista was good in HT29 and HTC-8 cells, whereas Clear^T2^ was not superior for any condition. Overall, 50% loss of signal intensity ranged from 85 to 133 μm of depth for DAPI and from 56 to 127 μm for DRAQ5 (Supplementary Table S3). 90% loss of signal intensity ranged from 151 to 327 μm of depth for DAPI and from 133 to 331 μm for DRAQ5 (Supplementary Table S3).

### Depth-Dependent Decline of Signal-to-Noise Ratio Varies With Cell Type

Next, SNR was calculated by the ratio of mean signal intensity and the corresponding standard deviation of the background. SNR values were then plotted as a function of normalized depth (Figures 4C,D) and the depth up to which the Rose criterion (SNR > 5) was met was determined (Supplementary Table S3). In HaCaT spheroids, maximal SNR values of 79 and 72 were reached for DAPI and DRAQ5 in the upper parts of spheroids, followed by more or less rapid decays. Similar as for signal intensities, the depth for reaching the Rose criterion was mostly the largest in Glycerol or Sca*l*eS samples, but also CytoVista showed good performance and was superior for DAPI in HaCaT, HT29, and HTC-8 cells, and for DRAQ5 in HaCaT cells (Supplementary Figure S5 and Supplementary Table S3). For DAPI, the normalized depths to reach Rose criterion ranged from 122 to 219 μm, for DRAQ5 from 233 to > 332 μm.

### Complexity of 3D-Cultivation Systems Affects Optical Clearing Efficiency

Although mono-culture spheroids are being used in many 3D-cell culture applications, more sophisticated approaches with different cell types in one model gain in importance. To evaluate the efficiency of tissue clearing for more complex co-cultures, we included a recently published tri-culture spheroid melanoma model (Klicks et al., 2019) as well as a chip-based microarray co-culture of fibroblasts and breast cancer cells into our analysis. In addition, these models allowed to test the influence of optical clearing on the performance and stability of frequently used markers in cell culture, i.e., CellTracker dyes and stably expressed fluorescent protein. First, we examined the clearing with the melanoma model. This consisted of a CCD-1137SK fibroblast core, surrounded by HaCaT keratinocytes and clusters of SK-MEL-28 melanoma cells, the latter two labeled with CellTracker Red and Green, respectively. Additionally, samples were stained with DAPI and anti-KI67 (Figure 5A). Embedding, optical clearing, and analyses were performed in analogy to mono-culture spheroids. Here, we observed similar post-fixation volume changes as for mono-culture spheroids, showing significantly reduced sample volumes for Mowiol, Clear^T2^, and CytoVista (Supplementary Figures S2, S3). For all tested methods, fluorescence of the different dyes was preserved, although to varying extent (Figure 5B). The DAPI signal was best preserved with CytoVista, followed by Mowiol and Glycerol. With respect to Rose criterion, Glycerol performed best (Supplementary Table S4), but none of the methods resulted in an absolute SNR cutoff depth higher than 89 μm (Supplementary Table S4). Notably, this value was much less than those obtained for mono-culture spheroids of HaCaT cells (153 μm, Supplementary Table S3) and CCD-1137SK fibroblasts (175 μm, Supplementary Table S3). For CellTracker Green, Mowiol worked best and none of the tested clearing methods could improve depth penetration for this dye. Likely, this was due to the primary spatial localization of CellTracker Green-labeled SK-MEL-28 cells in the peripheral region of the tri-cultures.

Next, we addressed optical clearing in microarray chip-based co-cultures of CCD-1137SK fibroblast and MDA-MB231-ECFP breast cancer cells. Here, cells were grown in collagen-coated cavities with a diameter of 300 μm and a depth of 200 μm. Fibroblasts and cancer cells were labeled with CellTracker Red and stably expressed ECFP, respectively. Additionally, cells were stained with DRAQ5 and anti-KI67 (Figures 6A,B). Except determination of clearing-induced volume changes and corresponding normalization of depth values, which were omitted, analyses were done as for spheroids. This revealed a particular sensitivity of ECFP to clearing. While Mowiol and Glycerol maintained ECFP signals, the other tested protocols led to a loss of signal intensity (Figure 6C). This was particularly pronounced for Clear^T2^, which abolished ECFP fluorescence almost completely. Signal loss was less dramatic for the other dyes, but overall only Mowiol, Sca*l*eS, and Glycerol yielded a balanced maintenance of fluorescence for all dyes. While Sca*l*eS and Glycerol led to an improvement of signal intensity in greater depth of the cavities, Mowiol performed badly in this aspect (Supplementary Table S4). The depth-dependent Rose criterion cutoff of DRAQ5 signals was met for Sca*l*eS and Glycerol at approximately 110 μm of depth (Figure 6D and Supplementary Table S4). In summary, these data revealed that complex 3D-cultivation systems pose an increased challenge for clearing protocols.

### Optical Clearing and z-Compensation Improve Data Segmentation

To evaluate the effects of clearing on quantitative image analysis of 3D-datasets, the segmentation efficiencies of the different clearing methods were compared. Therefore, DRAQ5-stained nuclei and KI67^+^ cells were segmented and counted for all samples. This revealed a high variance between different clearing protocols and cell lines (Figure 7 and Supplementary Figure S6). While nuclei in HaCaT spheroids could only be detected in the outer spheroid regions in PBS samples, Mowiol, Clear^T2^, Sca*l*eS, and Glycerol allowed improved segmentation of nuclei and KI67^+^ cells toward the spheroids core (Figure 7A). Correspondingly, quantitative data showed significantly more nuclei and KI67^+^ cells with all clearing methods except for CytoVista as compared to PBS controls (Figures 7B,C). Depending on the quality of signal preservation, depth penetration, and SNR, similarly enhanced segmentation results were also obtained for the other cell types (Supplementary Figure S6). Overall, the most consistent improvements were observed for glycerol and Sca*l*eS.

Notably, for all previous experiments, image acquisition was performed without depth-dependent adjustment of laser intensity or detection gain (z-compensation), in order to ensure comparability of methods. To test potential improvements of data quality and analysis by z-compensation, Glycerol-cleared HaCaT spheroids were imaged ± z-compensation by stepwise linear increase of excitation laser intensity, followed by quantification of depth-dependent signal intensity, SNR (Figure 8), and segmentation (Figure 9). Contrary to the data taken with static image acquisition parameters, z-compensation yielded almost constant levels for signal intensity (Figure 8B) and SNR throughout the central region of the entire spheroid (Figure 8C). This also affected segmentation of nuclei, which was clearly improved in deeper spheroid layers upon z-compensation (Figure 9A). As a consequence, counts of nuclei and KI67^+^ cells increased (Figures 9B,C). Likely, because cell proliferation was enriched in the peripheral spheroid layers, the rise in nuclei and KI67^+^ cell counts occurred in a disproportional manner, leading to an altered percentage of KI67^+^ positive cells (Figure 9D).

## Discussion

Recently, the increasing relevance of 3D-*in vitro* models in biomedical research has expanded the utilization of optical clearing methods from tissues and organs to 3D-cell cultures. However, investigations on cell type- and dye-dependent effects of optical clearing techniques have been sparse and not systematic. Ideally, a suitable optical clearing protocol should be easy to integrate into daily lab routine and manageable without specialized, cost intensive equipment or harmful chemicals. Moreover, sample integrity should be preserved and flexibility in the type of fluorescence labeling should be retained. Here, we compared simple clearing protocols with a range of cell types, dyes, and 3D culture conditions and found that soft clearing procedures such as RI-matching in Glycerol can work rather well for many applications.

### Effects of Clearing on Sample Shrinkage

Clearing-induced changes in sample volume can be desired in some cases. For example, expansion microscopy relies on increased sample dimensions to resolve objects that are normally below the optical resolution limit (Chen et al., 2015). However, this also leads to massive increase in time and resources needed for imaging, data processing and storage. In addition, expansion microscopy might affect sample or epitope integrity. On the other end, sample shrinkage might be advantageous for saving scanning and image processing time (Pan et al., 2016), but there is a risk of losing spatial resolution and segmentation of densely packed structures for automated quantification becomes more difficult (Jonkman et al., 2014). Thus, in most instances, preservation of sample volume is preferred for reasons of quantification, reproducibility, and structural integrity. Our results showed, that Sca*l*eS and Glycerol were able to maintain post-fixation sample size, whereas Mowiol, Clear^T2^, and CytoVista led to consistent further spheroid shrinkage, which was likely due to dehydration during tissue clearing (Pan et al., 2016). The strongest alteration of sample volume was observed with CytoVista followed by Clear^T2^. Previously, CytoVista was reported to cause only minor shrinkage. However, these studies referred to the clearing of tissues and not to small 3D structures such as spheroids (Hama et al., 2015; Ke et al., 2016; Bossolani et al., 2019). Also, with Clear^T2^, spheroids of human dermal fibroblasts (Costa et al., 2018b) and rodent-derived glioblastoma or neural stem cells (Dingle et al., 2015) showed no significant alterations of sample size. In contrast, constant monitoring of sample volume in an on-chip approach of optical clearing revealed an initial reduction of spheroid area by 20% (Grist et al., 2016). These diverse findings may result from different protocols employed for immunostainings, pointing toward the influences of sample processing on the outcome of sample volume. In contrast to Clear^T2^ and CytoVista, optical clearing by our modified version of Sca*l*eS did not alter the post-fixation sample volume. This is consistent with recent observations, where RI matching of spheroids used only Sca*l*eS4 solution (Boutin et al., 2018).

### Effects of Clearing on Dye Preservation and SNR

Further principal factors in the selection of an appropriate clearing protocol are preservation of fluorescence signal intensity and its accentuation against a background of autofluorescence, with the latter being particularly critical for segmentation purposes. While all tested procedures yielded analyzable signal intensities at the upper object layers for DAPI, DRAQ5, AlexaFluor488, and CellTracker dyes, ECFP fluorescence was compromised with Sca*l*eS and CytoVista and almost completely abolished by Clear^T2^. On tissue sections, this effect of Clear^T2^ was already observed for GFP and YFP and attributed to the formamide-induced non-aqueous microenvironment which is part of this method (Yu et al., 2016; Li et al., 2017). As for SNR, the maximal values were mostly higher for DRAQ5 than for DAPI regardless of cell type and clearing protocol. However, for both dyes, SNR decayed at a similar depth below the Rose criterion in non z-compensated image stacks. Importantly, z-compensation had a rather beneficial effect on keeping both, signal intensity and SNR, high over the entire spheroid range. This demonstrates, that proper z-compensation is a critical component for quantitative segmentation approaches and it suggests that SNR is dependent on fluorescence signal intensity even in confocal microscopy.

### Effects of Sample Complexity on Clearing Efficacy

Clearing of complex 3D-co-culture systems revealed, that optical clearing is becoming less effective with increasing sample intricacy. Compared to respective mono-culture spheroids, a tri-culture model of fibroblasts, keratinocytes, and peripheral melanoma cells showed decreased sample transparency and depth-dependent image quality for all tested clearing methods. Similarly, the chip-based co-cultures of fibroblast and breast cancer cells were penetrated in an incomplete manner. We suggest, that not only cell type-specific characteristics, but also the interaction between different cell types affects efficiency of optical clearing, most likely due to altered compactness (Desmaison et al., 2018) and additional scattering sources such as extracellular matrix (Senthebane et al., 2018). This is of particular importance when intracellular (e.g., keratins) or extracellular protein composition change during naturally occurring differentiation and stratification processes, like in skin 3D-models.

### Effects of Clearing on Segmentation

Precise segmentation of 3D microscopy data is fundamental to quantify changes between experimental groups at the single cell level, e.g., after treatment with a therapeutic agent. The image analysis pipeline used here combined a number of different pre-processing algorithms with a marker-controlled three-dimensional watershed segmentation approach that could reach an F-score of 0.88 (Schmitz et al., 2017). However, to obtain high segmentation accuracy, excellent raw data quality is of paramount importance (Roeder et al., 2012; Bassel, 2015). Notably, we observed gross differences in the number of segmented nuclei for the same samples cleared with distinct protocols. In addition, z-compensation during image acquisition proved to ultimately enhance data quality. Principal factors that determined image goodness were here found to be (i) high SNR, (ii) uniformity of SNR in xy and z, and (iii) appropriate resolution and packing of objects. Importantly, it is the combination of all three parameters that needs to be project-dependently optimized, because these will mutually affect each other.

Ad (i): as noted earlier, decent SNR values were obtained in the superficial layers of image acquisition for all clearing protocols and cell types. But even with the best clearing protocol, it tended to decay rapidly for all samples, if no z-compensation was used. Accordingly, data segmentation was accurate at the beginning of a data stack and deteriorated deeper within. Our analysis showed that segmentation was reliable at SNR around 20, while at the Rose criterion, i.e., SNR = 5, it was already insufficient. This does not necessarily mean that data with Rose level of SNR could not be properly analyzed. Indeed, we suggest that the algorithm used here would have still been able to detect most nuclei well, but given that the settings were optimized to segment robustly in a higher SNR range, the automated processing underperformed at lower SNR values. Thus, while a certain minimal SNR is needed, variability of SNR appeared to pose a critical major limitation, as well.
Ad (ii): a general challenge with the imaging of 3D biological specimens is the non-linear scattering of light. As observed also in this study, that leads to an exponential loss in signal intensity beyond a certain depth. Furthermore, since biological specimens have mostly round shapes, peripheral parts of these objects are covered by thinner layers of scattering material than central or core regions and hence signal quality and brightness is higher in the periphery than in the core. Z-compensation as used here, can result in overexposure of peripheral zones, but fortunately, at least for the present study it did not pose a problem. Conversely, z-compensation not only amended the loss of SNR in depth but also the variation of SNR within one optical layer from border to center. In conclusion, z-compensation is highly recommended to obtain higher quality image stacks with 3D-cell cultures.
Ad (iii): at high cell densities or low resolution, nuclei tend to overlap. Regularly, separation of such dense objects is a big challenge for segmentation algorithms (Jonkman et al., 2014). For example, segmentation based on a 3D-seeded watershed algorithm detects regional differences in brightness. Diffuse and incomplete edges due to poor image quality will impede accurate and consistent object detection, especially if the nuclear staining is not homogeneously distributed (Toyoshima et al., 2016). Sample shrinkage is a further factor that affects the segmentation accuracy adversely since the cell nuclei are more densely packed with more overlapping objects. This may lead to under-segmentation, and hence to a lower number of detected nuclei compared to the original sample (Blin et al., 2019). It is likely, that the high variance in cell numbers between different clearing protocols as observed in the present study is at least partially due to sample shrinkage in combination with high cell density.

## Conclusion

In summary, we showed that inherent characteristics of cell lines influence the outcome of optical clearing and that protocols should be chosen in a sample-dependent manner. Factors to consider include size, complexity and composition of 3D cultures. In our hands, the hyperhydrating method Sca*l*eS and the aqueous high-RI solution Glycerol provided the best results concerning their ability to preserve fluorescence of applied dyes and proteins, while maintaining sample integrity (Table 2). Furthermore, the choice of the clearing method and z-compensation also strongly influence quantitative image analysis and should be taken into account in experimental planning.

**TABLE 2:** Summarized evaluation table of all clearing methods and assessed parameters regardless of cell type-specific variations.

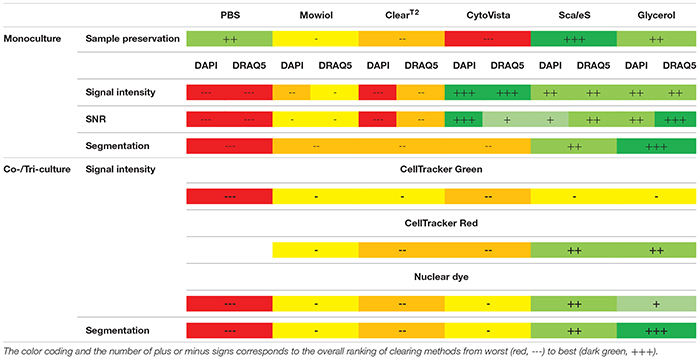

## Data Availability Statement

The datasets generated for this study are available on request to the corresponding author.

## Author Contributions

EN, MV, MH, and RR conceptualized and wrote the manuscript. EN, MV, JK, EM, TC, FK, and RB produced and analyzed experimental data. TE-F, KR, PS, TL, RS, and JM contributed materials, conceptual ideas, and participated in writing of the manuscript.

## Conflict of Interest

TE-F, KR, and PS were employed by the company B.R.A.I.N. AG. RS and JM were employed by the company Merck Healthcare KGaA. The remaining authors declare that the research was conducted in the absence of any commercial or financial relationships that could be construed as a potential conflict of interest.

## References

[B1] Alepee N. Bahinski A. Daneshian M. De Wever B. Fritsche E. Goldberg A. (2014). State-of-the-art of 3D cultures (organs-on-a-chip) in safety testing and pathophysiology. *ALTEX* 31 441–477. 10.14573/altex.1406111 25027500 PMC4783151

[B2] Ariel P. (2017). A beginner’s guide to tissue clearing. *Int. J. Biochem. Cell Biol.* 84 35–39. 10.1016/j.biocel.2016.12.009 28082099 PMC5336404

[B3] Bassel G. W. (2015). Accuracy in quantitative 3D image analysis. *Plant Cell* 27 950–953. 10.1105/tpc.114.135061 25804539 PMC4558689

[B4] Bauer S. Wennberg Huldt C. Kanebratt K. P. Durieux I. Gunne D. Andersson S. (2018). Functional coupling of human pancreatic islets and liver spheroids on-a-chip: towards a novel human ex vivo type 2 diabetes model. *Sci. Rep.* 8:1672. 10.1038/s41598-017-14815-w 29362490 PMC5780393

[B5] Berlanga M. L. Phan S. Bushong E. A. Wu S. Kwon O. Phung B. S. (2011). Three-dimensional reconstruction of serial mouse brain sections: solution for flattening high-resolution large-scale mosaics. *Front. Neuroanat.* 5:17. 10.3389/fnana.2011.00017 21629828 PMC3096995

[B6] Blin G. Sadurska D. Portero Migueles R. Chen N. Watson J. A. Lowell S. (2019). Nessys: a new set of tools for the automated detection of nuclei within intact tissues and dense 3D cultures. *PLoS Biol.* 17:e3000388. 10.1371/journal.pbio.3000388 31398189 PMC6703695

[B7] Bossolani G. D. P. Pintelon I. Detrez J. D. Buckinx R. Thys S. Zanoni J. N. (2019). Comparative analysis reveals Ce3D as optimal clearing method for in toto imaging of the mouse intestine. *Neurogastroenterol. Motil.* 31:e13560. 10.1111/nmo.13560 30761698

[B8] Boutin M. E. Voss T. C. Titus S. A. Cruz-Gutierrez K. Michael S. Ferrer M. (2018). A high-throughput imaging and nuclear segmentation analysis protocol for cleared 3D culture models. *Sci. Rep.* 8:11135. 10.1038/s41598-018-29169-0 30042482 PMC6057966

[B9] Carvalho M. R. Lima D. Reis R. L. Oliveira J. M. Correlo V. M. (2017). Anti-cancer drug validation: the contribution of tissue engineered models. *Stem Cell Rev. Rep.* 13 347–363. 10.1007/s12015-017-9720-x 28233276

[B10] Chen F. Tillberg P. W. Boyden E. S. (2015). Optical imaging. Expansion microscopy. *Science* 347 543–548. 10.1126/science.1260088 25592419 PMC4312537

[B11] Chen Y. Shen Q. White S. L. Gokmen-Polar Y. Badve S. Goodman L. J. (2019). Three-dimensional imaging and quantitative analysis in CLARITY processed breast cancer tissues. *Sci. Rep.* 9:5624. 10.1038/s41598-019-41957-w 30948791 PMC6449377

[B12] Chen Y. Tsai Y. H. Liu Y. A. Lee S. H. Tseng S. H. Tang S. C. (2013). Application of three-dimensional imaging to the intestinal crypt organoids and biopsied intestinal tissues. *ScientificWorldJournal* 2013:624342. 10.1155/2013/624342 24348177 PMC3848346

[B13] Chen Y. Y. Silva P. N. Syed A. M. Sindhwani S. Rocheleau J. V. Chan W. C. (2016). Clarifying intact 3D tissues on a microfluidic chip for high-throughput structural analysis. *Proc. Natl. Acad. Sci. U.S.A.* 113 14915–14920. 10.1073/pnas.1609569114 27956625 PMC5206515

[B14] Chiang A. S. Lin W. Y. Liu H. P. Pszczolkowski M. A. Fu T. F. Chiu S. L. (2002). Insect NMDA receptors mediate juvenile hormone biosynthesis. *Proc. Natl. Acad. Sci. U.S.A.* 99 37–42. 10.1073/pnas.012318899 11773617 PMC117510

[B15] Chiricozzi A. Romanelli M. Panduri S. Donetti E. Prignano F. (2017). Relevance of in vitro 3-D skin models in dissecting cytokine contribution to psoriasis pathogenesis. *Histol. Histopathol.* 32 893–898. 10.14670/HH-11-877 28124315

[B16] Chung K. Wallace J. Kim S. Y. Kalyanasundaram S. Andalman A. S. Davidson T. J. (2013). Structural and molecular interrogation of intact biological systems. *Nature* 497 332–337. 10.1038/nature12107 23575631 PMC4092167

[B17] Costa E. C. Moreira A. F. de Melo-Diogo D. Correia I. J. (2018a). ClearT immersion optical clearing method for intact 3D spheroids imaging through confocal laser scanning microscopy. *Opt. Laser Technol.* 106 94–99. 10.1016/j.optlastec.2018.04.002 29752799

[B18] Costa E. C. Moreira A. F. Melo-Diogo D. D. Correia I. J. (2018b). Polyethylene glycol molecular weight influences the clear. *J. Biomed. Opt.* 23 1–11. 10.1117/1.JBO.23.5.055003 29752799

[B19] Das V. Bruzzese F. Konecny P. Iannelli F. Budillon A. Hajduch M. (2015). Pathophysiologically relevant in vitro tumor models for drug screening. *Drug Discov. Today* 20 848–855. 10.1016/j.drudis.2015.04.004 25908576

[B20] Desmaison A. Guillaume L. Triclin S. Weiss P. Ducommun B. Lobjois V. (2018). Impact of physical confinement on nuclei geometry and cell division dynamics in 3D spheroids. *Sci. Rep.* 8:8785. 10.1038/s41598-018-27060-6 29884887 PMC5993719

[B21] Dingle Y.-T. L. Boutin M. E. Chirila A. M. Livi L. L. Labriola N. R. Jakubek L. M. (2015). Three-dimensional neural spheroid culture: an in vitro model for cortical studies. *Tissue Eng. Part C Methods* 21 1274–1283. 10.1089/ten.TEC.2015.0135 26414693 PMC4663656

[B22] Dodt H. U. Leischner U. Schierloh A. Jahrling N. Mauch C. P. Deininger K. (2007). Ultramicroscopy: three-dimensional visualization of neuronal networks in the whole mouse brain. *Nat. Methods* 4 331–336. 10.1038/nmeth1036 17384643

[B23] Drost J. Clevers H. (2018). Organoids in cancer research. *Nat. Rev. Cancer* 18 407–418. 10.1038/s41568-018-0007-6 29692415

[B24] Duval K. Grover H. Han L. H. Mou Y. Pegoraro A. F. Fredberg J. (2017). Modeling physiological events in 2d vs. 3d cell culture. *Physiology (Bethesda)* 32 266–277. 10.1152/physiol.00036.2016 28615311 PMC5545611

[B25] Erturk A. Becker K. Jahrling N. Mauch C. P. Hojer C. D. Egen J. G. (2012). Three-dimensional imaging of solvent-cleared organs using 3DISCO. *Nat. Protoc.* 7 1983–1995. 10.1038/nprot.2012.119 23060243

[B26] Fong E. L. Wan X. Yang J. Morgado M. Mikos A. G. Harrington D. A. (2016). A 3D in vitro model of patient-derived prostate cancer xenograft for controlled interrogation of in vivo tumor-stromal interactions. *Biomaterials* 77 164–172. 10.1016/j.biomaterials.2015.10.059 26599623 PMC4684431

[B27] Grist S. M. Nasseri S. S. Poon T. Roskelley C. Cheung K. C. (2016). On-chip clearing of arrays of 3-D cell cultures and micro-tissues. *Biomicrofluidics* 10:044107. 10.1063/1.4959031 27493703 PMC4958101

[B28] Grootjans J. Hundscheid I. H. Lenaerts K. Boonen B. Renes I. B. Verheyen F. K. (2013). Ischaemia-induced mucus barrier loss and bacterial penetration are rapidly counteracted by increased goblet cell secretory activity in human and rat colon. *Gut* 62 250–258. 10.1136/gutjnl-2011-301956 22637697

[B29] Hafner M. Niepel M. Subramanian K. Sorger P. K. (2017). designing drug-response experiments and quantifying their results. *Curr. Protoc. Chem. Biol.* 9 96–116. 10.1002/cpch.19 28628201 PMC5729909

[B30] Hama H. Hioki H. Namiki K. Hoshida T. Kurokawa H. Ishidate F. (2015). ScaleS: an optical clearing palette for biological imaging. *Nat. Neurosci.* 18 1518–1529. 10.1038/nn.4107 26368944

[B31] Hama H. Kurokawa H. Kawano H. Ando R. Shimogori T. Noda H. (2011). Scale: a chemical approach for fluorescence imaging and reconstruction of transparent mouse brain. *Nat. Neurosci.* 14 1481–1488. 10.1038/nn.2928 21878933

[B32] Hochheimer A. Krohn M. Rudert K. Riedel K. Becker S. Thirion C. (2014). Endogenous gustatory responses and gene expression profile of stably proliferating human taste cells isolated from fungiform papillae. *Chem. Senses* 39 359–377. 10.1093/chemse/bju009 24621663

[B33] Hou B. Zhang D. Zhao S. Wei M. Yang Z. Wang S. (2015). Scalable and DiI-compatible optical clearance of the mammalian brain. *Front. Neuroanat.* 9:19. 10.3389/fnana.2015.00019 25759641 PMC4338786

[B34] Hübner J. Raschke M. Rütschle I. Gräßle S. Hasenberg T. Schirrmann K. (2018). Simultaneous evaluation of anti-EGFR-induced tumour and adverse skin effects in a microfluidic human 3D co-culture model. *Sci. Rep.* 8:15010. 10.1038/s41598-018-33462-3 30301942 PMC6177413

[B35] Imamura Y. Mukohara T. Shimono Y. Funakoshi Y. Chayahara N. Toyoda M. (2015). Comparison of 2D- and 3D-culture models as drug-testing platforms in breast cancer. *Oncol. Rep.* 33 1837–1843. 10.3892/or.2015.3767 25634491

[B36] Jonkman J. Brown C. M. Cole R. W. (2014). Quantitative confocal microscopy: beyond a pretty picture. *Methods Cell Biol.* 123 113–134. 10.1016/B978-0-12-420138-5.00007-0 24974025

[B37] Kabadi P. K. Vantangoli M. M. Rodd A. L. Leary E. Madnick S. J. Morgan J. R. (2015). Into the depths: techniques for in vitro three-dimensional microtissue visualization. *Biotechniques* 59 279–286. 10.2144/000114353 26554505 PMC4804457

[B38] Ke M. T. Fujimoto S. Imai T. (2013). SeeDB: a simple and morphology-preserving optical clearing agent for neuronal circuit reconstruction. *Nat. Neurosci.* 16 1154–1161. 10.1038/nn.3447 23792946

[B39] Ke M. T. Nakai Y. Fujimoto S. Takayama R. Yoshida S. Kitajima T. S. (2016). Super-resolution mapping of neuronal circuitry with an index-optimized clearing agent. *Cell Rep.* 14 2718–2732. 10.1016/j.celrep.2016.02.057 26972009

[B40] Khodabukus A. Prabhu N. Wang J. Bursac N. (2018). In vitro tissue-engineered skeletal muscle models for studying muscle physiology and disease. *Adv. Healthc. Mater.* 7:e1701498. 10.1002/adhm.201701498 29696831 PMC6105407

[B41] Klicks J. Masslo C. Kluth A. Rudolf R. Hafner M. (2019). A novel spheroid-based co-culture model mimics loss of keratinocyte differentiation, melanoma cell invasion, and drug-induced selection of ABCB5-expressing cells. *BMC Cancer* 19:402. 10.1186/s12885-019-5606-4 31035967 PMC6489189

[B42] Kuwajima T. Sitko A. A. Bhansali P. Jurgens C. Guido W. Mason C. (2013). ClearT: a detergent- and solvent-free clearing method for neuronal and non-neuronal tissue. *Development* 140 1364–1368. 10.1242/dev.091844 23444362 PMC3912244

[B43] Lancaster M. A. Renner M. Martin C. A. Wenzel D. Bicknell L. S. Hurles M. E. (2013). Cerebral organoids model human brain development and microcephaly. *Nature* 501 373–379. 10.1038/nature12517 23995685 PMC3817409

[B44] Laugisch O. Cosgarea R. Nikou G. Nikolidakis D. Donos N. Salvi G. E. (2019). Histologic evidence of periodontal regeneration in furcation defects: a systematic review. *Clin. Oral. Investig.* 23 2861–2906. 10.1007/s00784-019-02964-3 31165313

[B45] Lee C. T. Bendriem R. M. Wu W. W. Shen R. F. (2017). 3D brain organoids derived from pluripotent stem cells: promising experimental models for brain development and neurodegenerative disorders. *J. Biomed. Sci.* 24:59. 10.1186/s12929-017-0362-8 28822354 PMC5563385

[B46] Leong A. S. (2004). Pitfalls in diagnostic immunohistology. *Adv. Anat. Pathol.* 11 86–93. 15090844 10.1097/00125480-200403000-00002

[B47] Li W. Germain R. N. Gerner M. Y. (2017). Multiplex, quantitative cellular analysis in large tissue volumes with clearing-enhanced 3D microscopy (Ce3D). *Proc. Natl. Acad. Sci. U.S.A.* 114 E7321–E7330. 10.1073/pnas.1708981114 28808033 PMC5584454

[B48] Luca A. C. Mersch S. Deenen R. Schmidt S. Messner I. Schafer K. L. (2013). Impact of the 3D microenvironment on phenotype, gene expression, and EGFR inhibition of colorectal cancer cell lines. *PLoS One* 8:e59689. 10.1371/journal.pone.0059689 23555746 PMC3608563

[B49] Marchevsky A. M. Wick M. R. (2015). Diagnostic difficulties with the diagnosis of small cell carcinoma of the lung. *Semin. Diagn. Pathol.* 32 480–488. 10.1053/j.semdp.2015.11.001 26597580

[B50] Masson A. Escande P. Frongia C. Clouvel G. Ducommun B. Lorenzo C. (2015). High-resolution in-depth imaging of optically cleared thick samples using an adaptive SPIM. *Sci. Rep.* 5:16898. 10.1038/srep16898 26576666 PMC4649629

[B51] Mertz J. (2011). Optical sectioning microscopy with planar or structured illumination. *Nat. Methods* 8 811–819. 10.1038/nmeth.1709 21959136

[B52] Murray E. Cho J. H. Goodwin D. Ku T. Swaney J. Kim S. Y. (2015). Simple, scalable proteomic imaging for high-dimensional profiling of intact systems. *Cell* 163 1500–1514. 10.1016/j.cell.2015.11.025 26638076 PMC5275966

[B53] Pan C. Cai R. Quacquarelli F. P. Ghasemigharagoz A. Lourbopoulos A. Matryba P. (2016). Shrinkage-mediated imaging of entire organs and organisms using uDISCO. *Nat. Methods* 13 859–867. 10.1038/nmeth.3964 27548807

[B54] Pereira J. F. Awatade N. T. Loureiro C. A. Matos P. Amaral M. D. Jordan P. (2016). The third dimension: new developments in cell culture models for colorectal research. *Cell. Mol. Life Sci.* 73 3971–3989. 10.1007/s00018-016-2258-2 27147463 PMC11108567

[B55] Renier N. Wu Z. Simon D. J. Yang J. Ariel P. Tessier-Lavigne M. (2014). iDISCO: a simple, rapid method to immunolabel large tissue samples for volume imaging. *Cell* 159 896–910. 10.1016/j.cell.2014.10.010 25417164

[B56] Renner M. Lancaster M. A. Bian S. Choi H. Ku T. Peer A. (2017). Self-organized developmental patterning and differentiation in cerebral organoids. *EMBO J.* 36 1316–1329. 10.15252/embj.201694700 28283582 PMC5430225

[B57] Richardson D. S. Lichtman J. W. (2015). Clarifying tissue clearing. *Cell* 162 246–257. 10.1016/j.cell.2015.06.067 26186186 PMC4537058

[B58] Roeder A. H. Cunha A. Burl M. C. Meyerowitz E. M. (2012). A computational image analysis glossary for biologists. *Development* 139 3071–3080. 10.1242/dev.076414 22872081

[B59] Roelofs A. J. De Bari C. (2019). Immunostaining of skeletal tissues. *Methods Mol. Biol.* 1914 437–450. 10.1007/978-1-4939-8997-3_25 30729481

[B60] Rohe I. Huttner F. J. Plendl J. Drewes B. Zentek J. (2018). Comparison of different histological protocols for the preservation and quantification of the intestinal mucus layer in pigs. *Eur. J. Histochem.* 62:2874. 10.4081/ejh.2018.2874 29569874 PMC5820525

[B61] Schindelin J. Arganda-Carreras I. Frise E. Kaynig V. Longair M. Pietzsch T. (2012). Fiji: an open-source platform for biological-image analysis. *Nat. Methods* 9 676–682. 10.1038/nmeth.2019 22743772 PMC3855844

[B62] Schmitt J. M. Kumar G. (1998). Optical scattering properties of soft tissue: a discrete particle model. *Appl. Opt.* 37 2788–2797. 10.1364/ao.37.002788 18273225

[B63] Schmitz A. Fischer S. C. Mattheyer C. Pampaloni F. Stelzer E. H. (2017). Multiscale image analysis reveals structural heterogeneity of the cell microenvironment in homotypic spheroids. *Sci. Rep.* 7:43693. 10.1038/srep43693 28255161 PMC5334646

[B64] Senthebane D. A. Jonker T. Rowe A. Thomford N. E. Munro D. Dandara C. (2018). The role of tumor microenvironment in chemoresistance: 3d extracellular matrices as accomplices. *Int. J. Mol. Sci.* 19:2861. 10.3390/ijms19102861 30241395 PMC6213202

[B65] Shroyer N. F. (2016). Tumor organoids fill the niche. *Cell Stem Cell* 18 686–687. 10.1016/j.stem.2016.05.020 27257754

[B66] Silvestri L. Costantini I. Sacconi L. Pavone F. S. (2016). Clearing of fixed tissue: a review from a microscopist’s perspective. *J. Biomed. Opt.* 21:081205. 10.1117/1.jbo.21.8.081205 27020691

[B67] Smyrek I. Stelzer E. H. (2017). Quantitative three-dimensional evaluation of immunofluorescence staining for large whole mount spheroids with light sheet microscopy. *Biomed. Opt. Express* 8 484–499. 10.1364/BOE.8.000484 28270962 PMC5330556

[B68] Susaki E. A. Tainaka K. Perrin D. Kishino F. Tawara T. Watanabe T. M. (2014). Whole-brain imaging with single-cell resolution using chemical cocktails and computational analysis. *Cell* 157 726–739. 10.1016/j.cell.2014.03.042 24746791

[B69] Tainaka K. Kuno A. Kubota S. I Murakami T. Ueda H. R. (2016). Chemical Principles in tissue clearing and staining protocols for whole-body cell profiling. *Annu. Rev. Cell Dev. Biol.* 32 713–741. 27298088 10.1146/annurev-cellbio-111315-125001

[B70] Toyoshima Y. Tokunaga T. Hirose O. Kanamori M. Teramoto T. Jang M. S. (2016). Accurate automatic detection of densely distributed cell nuclei in 3d space. *PLoS Comput. Biol.* 12:e1004970. 10.1371/journal.pcbi.1004970 27271939 PMC4894571

[B71] Tsai P. S. Kaufhold J. P. Blinder P. Friedman B. Drew P. J. Karten H. J. (2009). Correlations of neuronal and microvascular densities in murine cortex revealed by direct counting and colocalization of nuclei and vessels. *J. Neurosci.* 29 14553–14570. 10.1523/JNEUROSCI.3287-09.2009 19923289 PMC4972024

[B72] Wang C. Tang Z. Zhao Y. Yao R. Li L. Sun W. (2014). Three-dimensional in vitro cancer models: a short review. *Biofabrication* 6:022001. 10.1088/1758-5082/6/2/022001 24727833

[B73] Wenzel C. Riefke B. Grundemann S. Krebs A. Christian S. Prinz F. (2014). 3D high-content screening for the identification of compounds that target cells in dormant tumor spheroid regions. *Exp. Cell Res.* 323 131–143. 10.1016/j.yexcr.2014.01.017 24480576

[B74] Williams M. P. I. Rigon M. Straka T. Hörner S. J. Thiel M. Gretz N. (2019). A novel optical tissue clearing protocol for mouse skeletal muscle to visualize endplates in their tissue context. *Front. Cell. Neurosci.* 13:49. 10.3389/fncel.2019.00049 30873005 PMC6401545

[B75] Wu M. Swartz M. A. (2014). Modeling tumor microenvironments in vitro. *J. Biomech. Eng.* 136:021011. 10.1115/1.4026447 24402507 PMC4023667

[B76] Xu J. Ma Y. Yu T. Zhu D. (2019). Quantitative assessment of optical clearing methods in various intact mouse organs. *J. Biophotonics* 12:e201800134. 10.1002/jbio.201800134 30318789

[B77] Yang B. Treweek J. B. Kulkarni R. P. Deverman B. E. Chen C.-K. Lubeck E. (2014). Single-cell phenotyping within transparent intact tissue through whole-body clearing. *Cell* 158 945–958. 10.1016/j.cell.2014.07.017 25088144 PMC4153367

[B78] Yu T. Qi Y. Wang J. Feng W. Xu J. Zhu J. (2016). Rapid and prodium iodide-compatible optical clearing method for brain tissue based on sugar/sugar-alcohol. *J. Biomed. Opt.* 21:081203. 10.1117/1.JBO.21.8.081203 26968577

[B79] Zschenker O. Streichert T. Hehlgans S. Cordes N. (2012). Genome-wide gene expression analysis in cancer cells reveals 3D growth to affect ECM and processes associated with cell adhesion but not DNA repair. *PLoS One* 7:e34279. 10.1371/journal.pone.0034279 22509286 PMC3324525

