# Supplementary figures and images for: Routine Optical Clearing of 3D-Cell Cultures: Simplicity Forward
Source: Front Mol Biosci. 2020 Feb 21;7:20. doi: 10.3389/fmolb.2020.00020 (PMC7046628; doi:10.3389/fmolb.2020.00020)

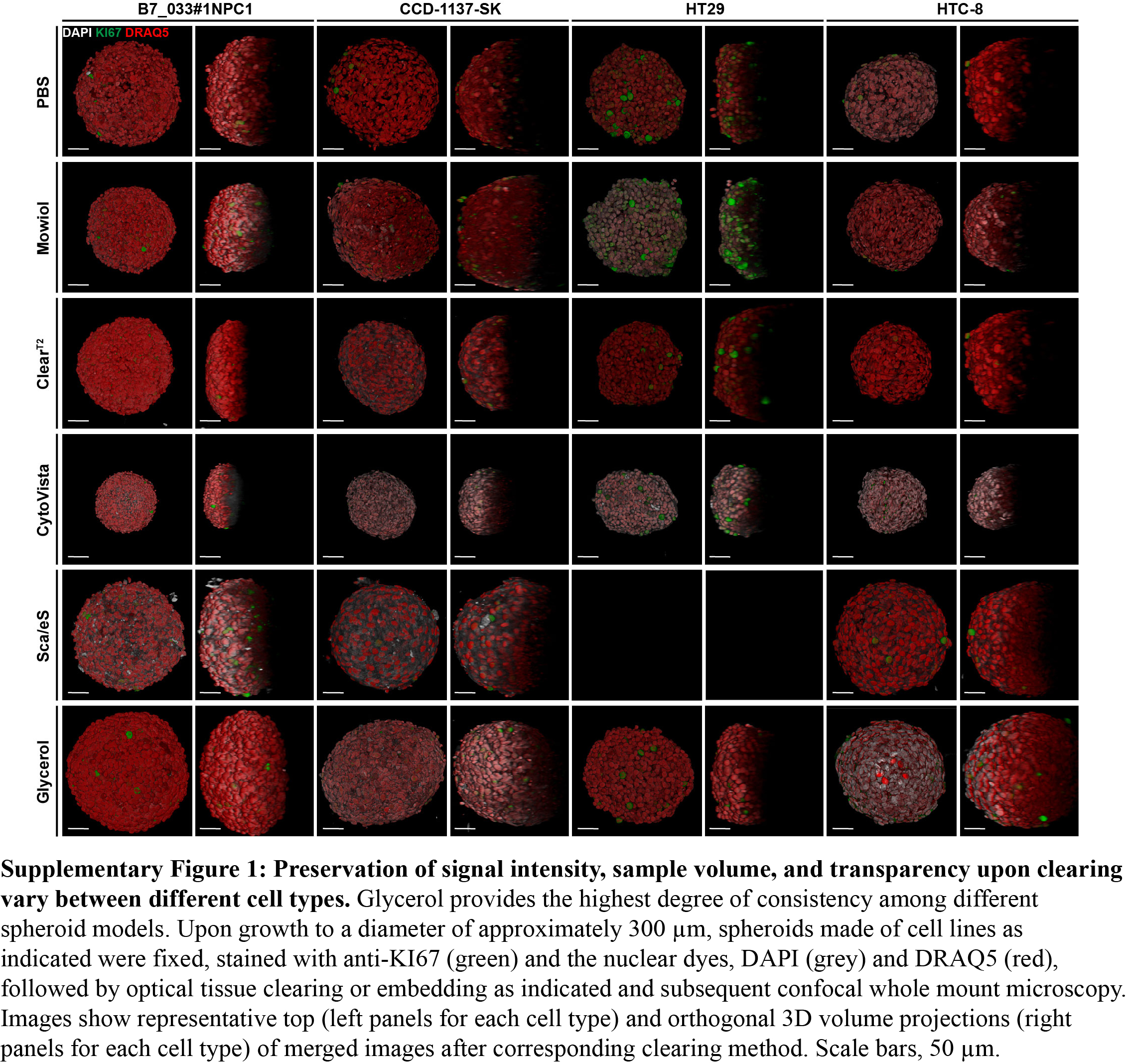

Supplement: Supplementary file 1 [file Image_1.JPEG]

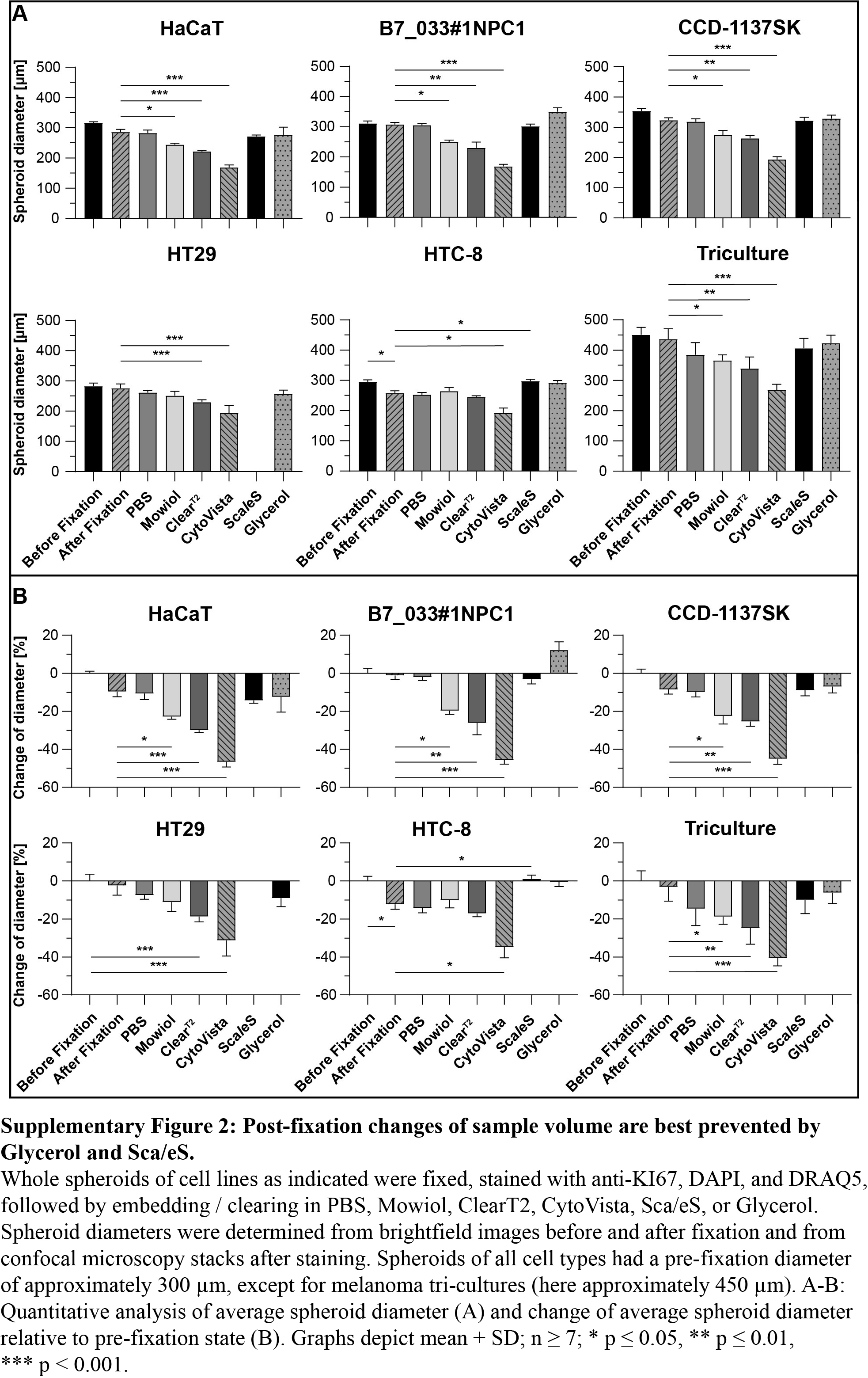

Supplement: Supplementary file 2 [file Image_2.JPEG]

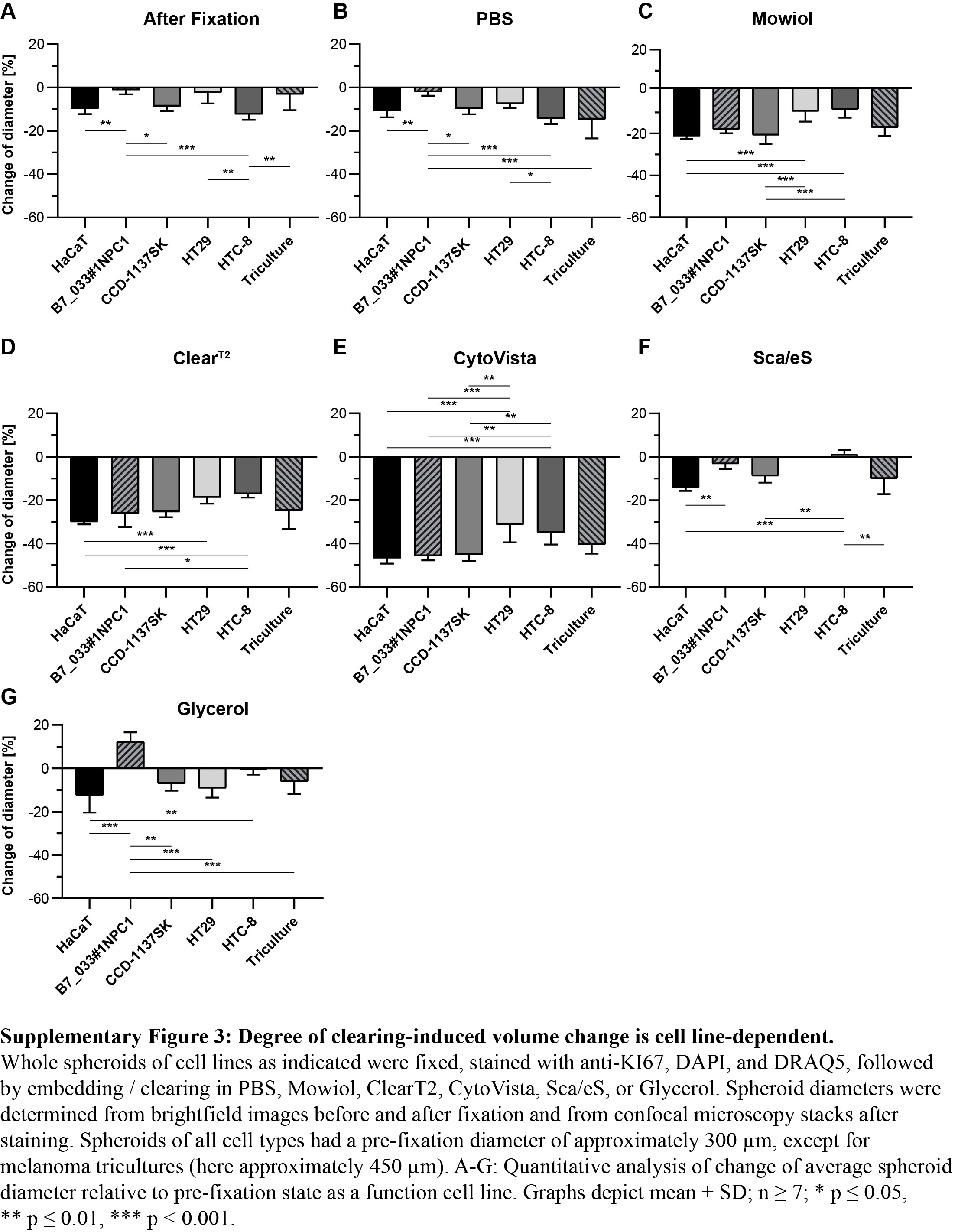

Supplement: Supplementary file 3 [file Image_3.JPEG]

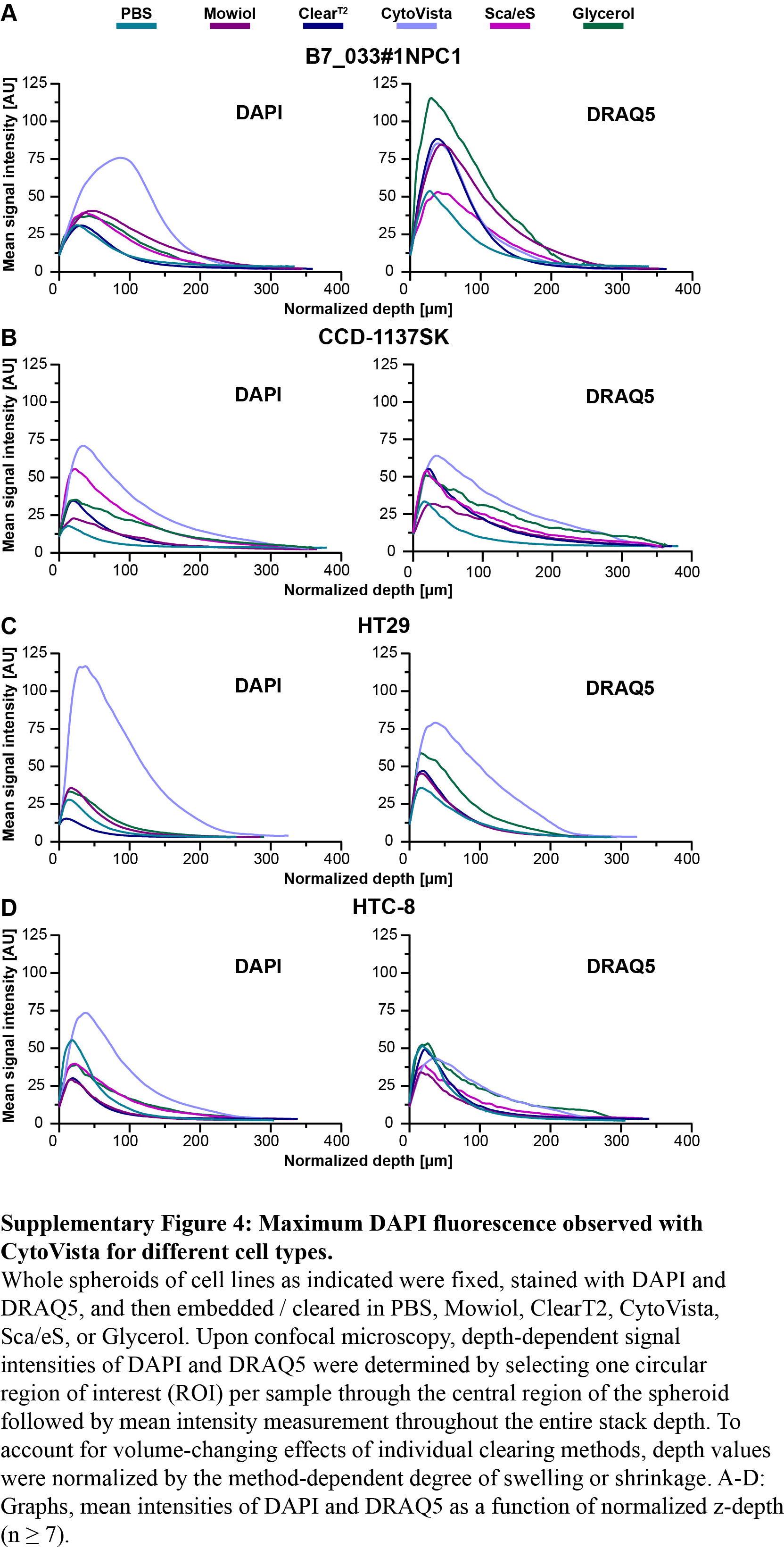

Supplement: Supplementary file 4 [file Image_4.JPEG]

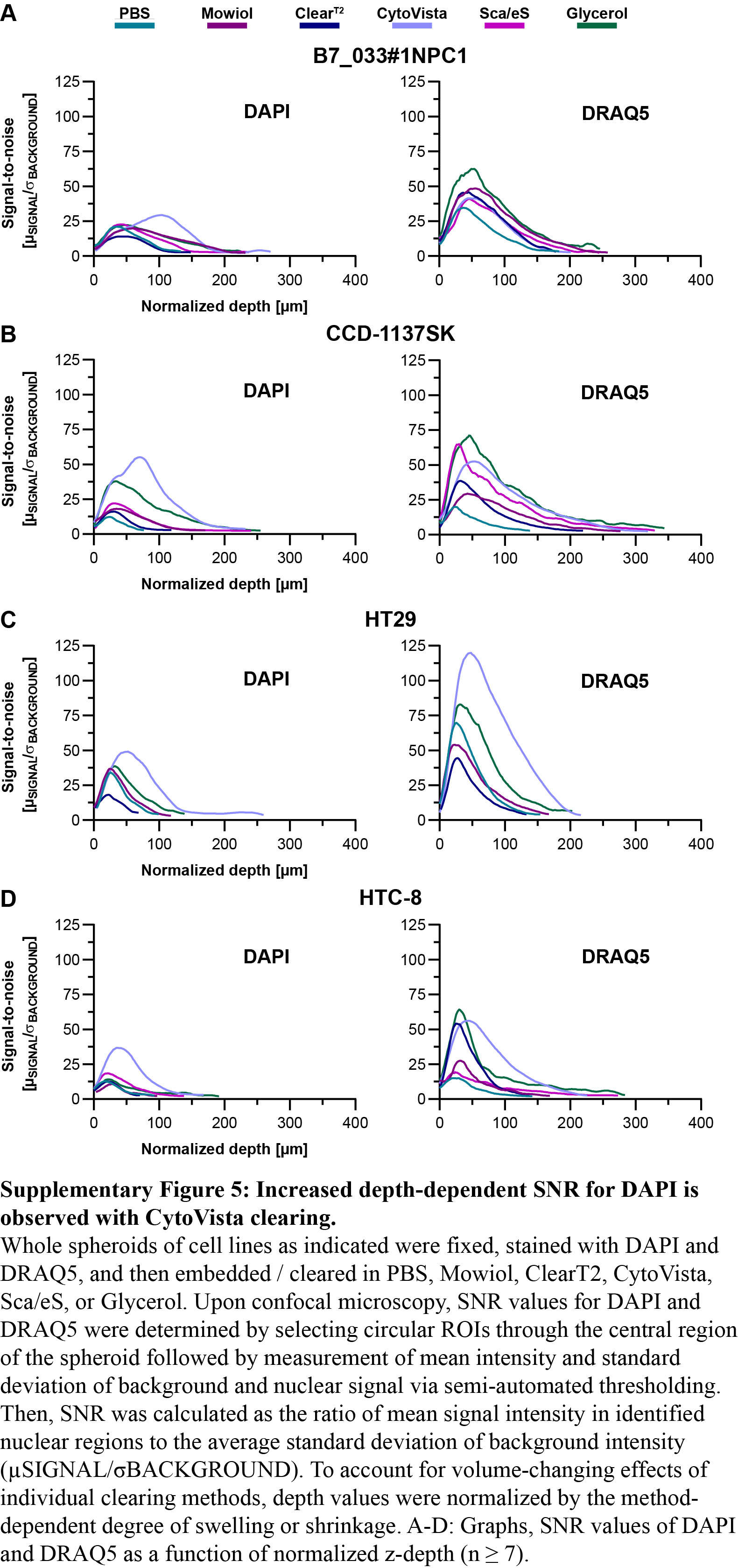

Supplement: Supplementary file 5 [file Image_5.JPEG]

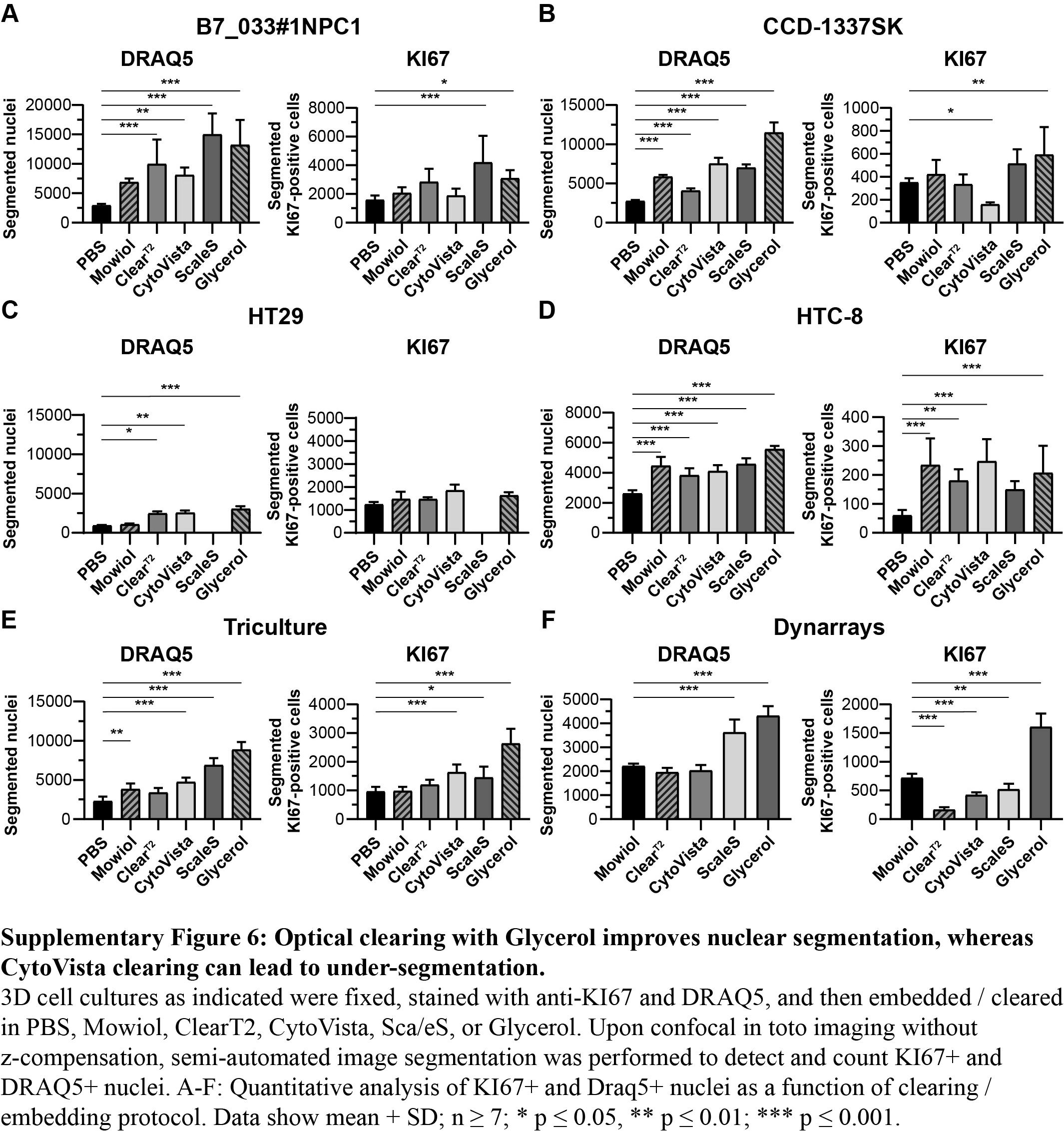

Supplement: Supplementary file 6 [file Image_6.JPEG]
