# Supplementary material for: Routine Optical Clearing of 3D-Cell Cultures: Simplicity Forward
Source: Front Mol Biosci. 2020 Feb 21;7:20. doi: 10.3389/fmolb.2020.00020 (PMC7046628; doi:10.3389/fmolb.2020.00020)
Supplement: Supplementary file 7 [file Table_1.DOCX]

| **Reagent** | **Supplier** | **Ordering No.** | **Lot No.** |
| --- | --- | --- | --- |
| **Cell culture media and reagents** |  |  |  |
| 10x Trypsin-EDTA solution | Sigma-Aldrich | 59418C | SLCC3240 |
| B27 Supplement (50x), serum free | Invitrogen | 17504044 | 1987565 |
| Basal Iscove Medium | Biochrome | F0465 | 1173E |
| Collagen 1 from rat tail tendon | Sigma-Aldrich | 11179179001 | 38429220 |
| DMEM | Capricorn | DMEM-HPA | CP18-2096 |
| DMEM/Ham's F-12, w/ GlutaMAX supplement | Invitrogen | 10565018 | 2124955 |
| Fetal calf serum (FCS) | Capricorn | FBS-12A | CP16-1422 |
| Gentamicine | Biochrome | A2712 | 950302 |
| GlutaMAX | Invitrogen | 35050061 | 2063464 |
| IMDM | Sigma-Aldrich | I3390 | RNBG9989 |
| Insulin | Sigma-Aldrich | I-6634 | 74H00845 |
| L-Glutamine (200 mM) | Capricorn | GLN-B | CP17-1801 |
| McCoy´s 5A | Capricorn | MCC-A | CP19-2689 |
| MCDB | Biochrome | F8105 | 0674G |
| Minimum Essential Medium Nonessential Amino Acids (MEM-NEAA) | Invitrogen | 10370070 | 2026977 |
| N-2 Supplement (100x) | Invitrogen | 17502048 | 1911689 |
| Pen/Strep (10.000 U penicillin, 10 mg/mL streptomycin) | Sigma-Aldrich | P4333 | 049M4857V |
| Recombinant human FGF-2 Type 147 | Cell Guidance Systems | GFH28-100 | 0313 |
| TrypLE Express Enzyme (1X), no phenol red | Invitrogen | 12604021 | 2085298 |
| **Transfection Reagents** |  |  |  |
| 5 % AseI enzyme | New England Biolabs | R0526S | 131701 |
| Alkaline phosphatase | Invitrogen | 100012546 | 1752270 |
| AseI buffer 3.1 | New England Biolabs | B7203S | 421612 |
| **Other chemicals and reagents** |  |  |  |
| Albumin Fraktion V (BSA) | Carl Roth | 8076.3 | 69279339 |
| CytoVista 3D Cell Culture Clearing Reagent | Invitrogen | V11315 | 9QZ53 |
| Dimethylsulfoxid (DMSO) | Carl Roth | A994.1 | 199282083 |
| D-sorbitol | Sigma-Aldrich | 1077581000 | M529358845 |
| Formamide | Sigma-Aldrich | 47671 | 372879/1 44997 |
| Glycerol | Carl Roth | 3783.2 | 237259780 |
| Glycine | Carl Roth | 3908.2 | 177257836 |
| Heparin sodium salt from porcine intestinal mucosa | Sigma-Aldrich | H3149-250KU | SLBN6012V |
| Mowiol 4-88 | Sigma-Aldrich | 713.2 | 269278961 |
| Paraformaldehyd | Carl Roth | 335.3 | 259284291 |
| Polyethylenglykol 8000 (PEG) | Sigma-Aldrich | P5413 | BCCC6496 |
| Triton X-100 | Carl Roth | T9284 | 19K01512 |
| Tween-20 | Sigma-Aldrich | P9416-50ML | 125K01031 |
| Urea | Sigma-Aldrich | 1084871000 | K40676887002 |
| **Dyes & Antibodies** |  |  |  |
| Anti-KI67 Antibody (rabbit polyclonal) | Sigma-Aldrich | AB9260 | 3195946 |
| CellTracker Green CMFDA Dye | ThermoFisher Scientific | C2925 | 1781143 |
| CellTracker Red CMPTX Dye | ThermoFisher Scientific | C34552 | 1890538 |
| DAPI dihydrochloride | Sigma-Aldrich | D9542-5MG | 28114320 |
| Donkey anti-Rabbit IgG (H+L) Alexa Fluor Plus 488 | ThermoFisher Scientific | A32790 | TI271741 |
| Donkey anti-Rabbit IgG (H+L) Alexa Fluor Plus 647 | ThermoFisher Scientific | A32795 | TJ271043 |
| DRAQ5 Fluorescent Probe Solution (5 mM) | ThermoFisher Scientific | 62252 | 511DR50200 |

Supplementary Table 1: Chemicals and reagents that were used for this study
