# Supplementary material for: Routine Optical Clearing of 3D-Cell Cultures: Simplicity Forward
Source: Front Mol Biosci. 2020 Feb 21;7:20. doi: 10.3389/fmolb.2020.00020 (PMC7046628; doi:10.3389/fmolb.2020.00020)
Supplement: Supplementary file 8 [file Table_2.docx]

| **DAPI** | | | | | | | | | |
| --- | --- | --- | --- | --- | --- | --- | --- | --- | --- |
|  | **Depth [µm]** | **25** | **50** | **75** | **100** | **125** | **150** | **175** | **200** |
| **PBS** | **% of darkening** | 21.55 | 62.33 | 76.67 | 80.56 | 82.05 | 82.66 | 83.51 | 100 |
|  | **SD [%]** | 15.05 | 8.25 | 2.31 | 0.73 | 0.42 | 0.83 | 0.29 | 0 |
| **Mowiol** | **% of darkening** | 18.08 | 59.85 | 81.99 | 89.52 | 92.06 | 93.10 | 93.67 | 94.06 |
|  | **SD [%]** | 7.44 | 5.99 | 2.88 | 1.47 | 0.86 | 1.03 | 0.98 | 0.71 |
| **Clear^T2^** | **% of darkening** | 13.87 | 60.90 | 80.54 | 86.54 | 88.74 | 89.88 | 100 | 100 |
|  | **SD [%]** | 4.77 | 1.78 | 0.51 | 0.27 | 0.16 | 0.11 | 0 | 0 |
| **CytoVista** | **% of darkening** | 5.89 | 48.01 | 69.51 | 80.65 | 91.05 | 100 | 100 | 100 |
|  | **SD [%]** | 14.12 | 9.17 | 5.88 | 3.40 | 3.27 | 0 | 0 | 0 |
| **Sca*l*eS** | **% of darkening** | 3.44 | 8.54 | 37.04 | 59.91 | 73.80 | 83.02 | 88.14 | 90.38 |
|  | **SD [%]** | 14.77 | 10.74 | 7.66 | 4.22 | 2.85 | 1.75 | 0.98 | 0.71 |
| **Glycerol** | **% of darkening** | 3.73 | 25.16 | 50.88 | 68.77 | 79.06 | 85.03 | 87.85 | 88.26 |
|  | **SD [%]** | 10.81 | 8.54 | 7.21 | 6.15 | 5.48 | 5.55 | 6.34 | 7.77 |
| **DRAQ5** | | | | | | | | | |
|  | **Depth [µm]** | **25** | **50** | **75** | **100** | **125** | **150** | **175** | **200** |
| **PBS** | **% of darkening** | 14.72 | 56.42 | 77.53 | 85.64 | 88.44 | 89.54 | 90.14 | 100 |
|  | **SD [%]** | 27.40 | 15.45 | 6.32 | 2.32 | 0.89 | 0.46 | 0.36 | 0 |
| **Mowiol** | **% of darkening** | 15.42 | 49.38 | 73.52 | 85.54 | 91.34 | 94.08 | 95.73 | 96.41 |
|  | **SD [%]** | 6.65 | 7.39 | 3.65 | 2.68 | 1.32 | 0.66 | 0.57 | 0.51 |
| **Clear^T2^** | **% of darkening** | 7.96 | 52.97 | 78.93 | 89.30 | 92.95 | 94.96 | 100 | 100 |
|  | **SD [%]** | 30.54 | 15.53 | 6.89 | 3.44 | 2.25 | 1.85 | 0 | 0 |
| **CytoVista** | **% of darkening** | 1.98 | 32.87 | 56.19 | 73.60 | 89.32 | 86.46 | 100 | 100 |
|  | **SD [%]** | 20.65 | 11.87 | 6.48 | 2.85 | 5.47 | 0 | 0 | 0 |
| **Sca*l*eS** | **% of darkening** | 0 | 15.83 | 42.47 | 59.44 | 70.33 | 77.91 | 83.09 | 86.18 |
|  | **SD [%]** | 12.67 | 7.41 | 5.82 | 4.19 | 2.62 | 2.46 | 1.07 | 1.35 |
| **Glycerol** | **% of darkening** | 8.18 | 25.84 | 47.51 | 62.50 | 73.38 | 80.13 | 84.78 | 86.55 |
|  | **SD [%]** | 13.39 | 13.20 | 8.06 | 5.95 | 3.61 | 3.60 | 5.84 | 9.16 |

Supplementary Table 2: Percentage loss of depth-dependent signal intensity in cleared HaCaT spheroids. Upon growth to a diameter of approximately 300 µm, HaCaT spheroids were fixed, stained with DAPI and DRAQ5, followed by optical clearing or embedding as indicated, and subsequent confocal whole mount microscopy without z-compensation. The Table reports the mean percentage loss of signal intensity with corresponding standard deviation (SD), calculated from the individual maximum signal intensity of each condition, at absolute imaging depths of 25-200 µm. N ≥ 7.
