# Supplementary material for: Routine Optical Clearing of 3D-Cell Cultures: Simplicity Forward
Source: Front Mol Biosci. 2020 Feb 21;7:20. doi: 10.3389/fmolb.2020.00020 (PMC7046628; doi:10.3389/fmolb.2020.00020)
Supplement: Supplementary file 9 [file Table_3.DOCX]

| **B7_033#1NPC1** | | | | | | | | | | | | |
| --- | --- | --- | --- | --- | --- | --- | --- | --- | --- | --- | --- | --- |
|  | **DAPI** | | | | | | **DRAQ5** | | | | | |
|  | **Absolute depth [µm]** | | | **Normalized depth [µm]** | | | **Absolute depth [µm]** | | | **Normalized depth [µm]** | | |
|  | 50% signal loss | 90%  signal loss | SNR < 5 | 50%  signal loss | 90%  signal loss | SNR < 5 | 50%  signal loss | 90%  signal loss | SNR < 5 | 50%  signal loss | 90%  signal loss | SNR < 5 |
| PBS | 75,0 | -- | 103,5 | 76,6 | -- | 105,7 | 78,0 | 216,0 | 139,5 | 79,6 | 220,5 | 142,4 |
| Mowiol | 111,0 | 220,5 | 154,5 | 138,2 | 274,5 | 192,4 | 94,5 | 190,5 | 171,0 | 117,7 | **237,2** | 212,9 |
| Clear^T2^ | 60,0 | 153,0 | 73,5 | 81,3 | 207,2 | 99,5 | 64,5 | 117,0 | 123,0 | 87,4 | 158,4 | 166,6 |
| CytoVista | 78,0 | 118,5 | 96,0 | **143,5** | 218,0 | 176,6 | 49,5 | 99,0 | 85,5 | 91,1 | 182,1 | 157,3 |
| Sca/eS | 102,0 | 214,5 | 133,5 | 105,4 | **221,6** | 137,9 | 114,0 | 211,5 | 187,5 | **117,8** | 218,5 | 193,7 |
| Glycerol | **133,5** | **237,0** | **201,0** | 118,9 | 211,1 | **179,0** | **123,0** | **226,5** | **265,5** | 109,6 | 201,8 | **236,5** |
| **HaCaT** | | | | | | | | | | | | |
|  | **DAPI** | | | | | | **DRAQ5** | | | | | |
|  | **Absolute depth [µm]** | | | **Normalized depth [µm]** | | | **Absolute depth [µm]** | | | **Normalized depth [µm]** | | |
|  | 50% signal loss | 90%  signal loss | SNR < 5 | 50%  signal loss | 90%  signal loss | SNR < 5 | 50%  signal loss | 90%  signal loss | SNR < 5 | 50%  signal loss | 90%  signal loss | SNR < 5 |
| PBS | 40,5 | -- | 64,5 | 45,4 | -- | 72,2 | 45,0 | 156,0 | 87,0 | 50,4 | 174,7 | 97,4 |
| Mowiol | 43,5 | 103,5 | 69,0 | 56,3 | 134,1 | 89,4 | 49,5 | 117,0 | 105,0 | 64,1 | 151,6 | 136,0 |
| Clear^T2^ | 42,0 | 154,5 | 61,5 | 60,0 | 220,6 | 87,8 | 48,0 | 103,5 | 96,0 | 68,5 | 147,8 | 137,1 |
| CytoVista | 51,0 | 121,5 | 117,0 | 95,6 | **227,7** | **219,3** | 66,0 | 126,0 | 117,0 | **123,7** | 236,2 | 219,3 |
| Sca/eS | **88,5** | **189,0** | **153,0** | **103,2** | 220,4 | 178,4 | **84,0** | **232,5** | **205,5** | 98,0 | **271,2** | **239,7** |
| Glycerol | 73,5 | -- | 142,5 | 84,0 | -- | 162,8 | 78,0 | -- | 195,0 | 89,1 | -- | 222,8 |
| **CCD-1337SK** | | | | | | | | | | | | |
|  | **DAPI** | | | | | | **DRAQ5** | | | | | |
|  | **Absolute depth [µm]** | | | **Normalized depth [µm]** | | | **Absolute depth [µm]** | | | **Normalized depth [µm]** | | |
|  | 50% signal loss | 90%  signal loss | SNR < 5 | 50%  signal loss | 90%  signal loss | SNR < 5 | 50%  signal loss | 90%  signal loss | SNR < 5 | 50%  signal loss | 90%  signal loss | SNR < 5 |
| PBS | 49,5 | -- | 51,0 | 54,9 | -- | 56,6 | 52,5 | -- | 85,5 | 58,2 | -- | 94,9 |
| Mowiol | 75,0 | -- | 85,5 | 96,7 | -- | 110,3 | 103,5 | 276,0 | 154,5 | 133,5 | 355,9 | 199,2 |
| Clear^T2^ | 48,0 | 222,0 | 54,0 | 64,4 | 297,7 | 72,4 | 58,5 | 199,5 | 120,0 | 78,4 | 267,5 | 160,9 |
| CytoVista | 60,0 | 156,0 | 105,0 | 109,0 | 283,5 | **190,8** | 73,5 | 171,0 | 142,5 | 133,6 | 310,7 | 258,9 |
| Sca/eS | 84,0 | 244,5 | 102,0 | 92,2 | 268,3 | 111,9 | 78,0 | 303,0 | 243,0 | 85,6 | 332,5 | 266,7 |
| Glycerol | **123,0** | **327,0** | **175,5** | **132,3** | **351,7** | 188,8 | **127,5** | **331,5** | **309,0** | **137,1** | **356,6** | **332,4** |
| **HT29** | | | | | | | | | | | | |
|  | **DAPI** | | | | | | **DRAQ5** | | | | | |
|  | **Absolute depth [µm]** | | | **Normalized depth [µm]** | | | **Absolute depth [µm]** | | | **Normalized depth [µm]** | | |
|  | 50% signal loss | 90%  signal loss | SNR < 5 | 50%  signal loss | 90%  signal loss | SNR < 5 | 50%  signal loss | 90%  signal loss | SNR < 5 | 50%  signal loss | 90%  signal loss | SNR < 5 |
| PBS | 31,5 | -- | 53,5 | 50,3 | -- | 85,9 | 39,5 | 115,5 | 81,5 | 63,2 | 186,5 | 131,4 |
| Mowiol | 34,5 | 122,5 | 58,5 | 57,4 | 205,8 | 97,9 | 43,5 | **133,5** | 89,5 | 72,5 | **224,4** | 150,2 |
| Clear^T2^ | 27,5 | -- | 37,5 | 49,8 | -- | 68,3 | 34,5 | 102,5 | 68,5 | 62,8 | 188,3 | 125,5 |
| CytoVista | **50,5** | 96,5 | 70,5 | **109,1** | 209,4 | **152,7** | **56,5** | 100,5 | **92,5** | **122,2** | 218,1 | **200,7** |
| Sca/eS |  |  |  |  |  |  |  |  |  |  |  |  |
| Glycerol | 43,5 | **151,5** | **81,5** | 70,9 | **249,1** | 133,6 | 49,5 | 121,5 | **Full Range** | 80,8 | 199,6 | **Full Range** |
| **HTC-8** | | | | | | | | | | | | |
|  | **DAPI** | | | | | | **DRAQ5** | | | | | |
|  | **Absolute depth [µm]** | | | **Normalized depth [µm]** | | | **Absolute depth [µm]** | | | **Normalized depth [µm]** | | |
|  | 50% signal loss | 90%  signal loss | SNR < 5 | 50%  signal loss | 90%  signal loss | SNR < 5 | 50%  signal loss | 90%  signal loss | SNR < 5 | 50%  signal loss | 90%  signal loss | SNR < 5 |
| PBS | 45,0 | 112,5 | 45,0 | 52,5 | 131,2 | 52,5 | 46,5 | 124,5 | 64,5 | 54,2 | 145,2 | 75,2 |
| Mowiol | 51,0 | 168,0 | 40,5 | 56,8 | 187,0 | 45,1 | 58,5 | 184,5 | 102,0 | 65,1 | 205,4 | 113,5 |
| Clear^T2^ | 43,5 | -- | 42,0 | 52,5 | -- | 50,6 | 52,5 | 150,0 | 96,0 | 63,3 | 180,9 | 115,8 |
| CytoVista | 63,0 | 145,5 | 79,5 | **96,7** | 223,3 | **122,0** | 75,0 | 162,0 | 129,0 | **115,1** | 248,6 | 198,0 |
| Sca/eS | **85,5** | **259,5** | **88,5** | 84,5 | 256,6 | 87,5 | 81,0 | **312,0** | 162,0 | 80,1 | **308,5** | 160,2 |
| Glycerol | 84,0 | 264,0 | 85,5 | 84,5 | **265,4** | 86,0 | **85,5** | 276,0 | **232,5** | 86,0 | 277,5 | **233,7** |

Supplementary Table 3: Overview of fluorescence penetration and SNR in depth of simple spheroids sorted by cell type. Upon growth to a diameter of approximately 300 µm, spheroids made of cell lines as indicated were fixed, stained with DAPI and DRAQ5, followed by optical tissue clearing or embedding as indicated and subsequent confocal whole mount microscopy without z-compensation. The table reports the average values for absolute and normalized depth of 50 % signal loss, 90 % signal loss, and SNR < 5 for DAPI and DRAQ5 in the absence of z-compensation. N ≥ 7. For clarity, maximal values are depicted with green shading.
