# Supplementary material for: Routine Optical Clearing of 3D-Cell Cultures: Simplicity Forward
Source: Front Mol Biosci. 2020 Feb 21;7:20. doi: 10.3389/fmolb.2020.00020 (PMC7046628; doi:10.3389/fmolb.2020.00020)
Supplement: Supplementary file 10 [file Table_4.DOCX]

| **Triculture** | | | | | | | | | | | | |
| --- | --- | --- | --- | --- | --- | --- | --- | --- | --- | --- | --- | --- |
|  | **DAPI** | | | | | | **CellTracker Green** | | | | | |
|  | **Absolute depth [µm]** | | | **Normalized depth [µm]** | | | **Absolute depth [µm]** | | | **Normalized depth [µm]** | | |
|  | 50% signal loss | 90%  signal loss | SNR  < 5 | 50%  signal loss | 90%  signal loss | SNR < 5 | 50%  signal loss | 90%  signal loss | SNR  < 5 | 50%  signal loss | 90%  signal loss | SNR  < 5 |
| PBS | 25,5 | 90,5 | 34,5 | 43,9 | 158,1 | 59,7 | 27,5 | -- | -- | 47,4 | -- | -- |
| Mowiol | 38,5 | 81,5 | 78,5 | 70,2 | 149,6 | 144,1 | 30,5 | -- | -- | 55,4 | -- | -- |
| Clear^T2^ | 30,5 | 80,5 | 47,5 | 59,8 | 159,5 | 93,7 | 28,5 | -- | -- | 55,8 | -- | -- |
| CytoVista | 33,5 | 71,5 | 59,5 | **83,1** | 178,9 | 148,6 | 21,5 | -- | -- | 52,9 | -- | -- |
| Sca/eS | 48,5 | **168,5** | 67,5 | 80,0 | **279,9** | 111,6 | 33,5 | -- | -- | 55,0 | -- | -- |
| Glycerol | **49,5** | 128,5 | **88,5** | 78,3 | 204,5 | **140,6** | 34,5 | -- | -- | 54,3 | -- | -- |
| **Dynarrays** | | | | | | | | | | | | |
|  | **CellTracker Red** | | | | | | **DRAQ5** | | | | | |
|  | **Absolute depth [µm]** | | | **Normalized depth [µm]** | | | **Absolute depth [µm]** | | | **Normalized depth [µm]** | | |
|  | 50% signal loss | 90%  signal loss | SNR < 5 | 50%  signal loss | 90%  signal loss | SNR < 5 | 50%  signal loss | 90%  signal loss | SNR < 5 | 50%  signal loss | 90%  signal loss | SNR < 5 |
| PBS | -- | -- | -- | -- | -- | -- | -- | -- | -- | -- | -- | -- |
| Mowiol | 58,5 | -- | -- | -- | -- | -- | 56,5 |  | 74,5 | -- | -- | -- |
| Clear^T2^ | 47,5 | -- | -- | -- | -- | -- | 44,5 | -- | 64,5 | -- | -- | -- |
| CytoVista | 42,5 | -- | -- | -- | -- | -- | 43,5 | -- | 45,5 | -- | -- | -- |
| Sca/eS | **92,5** | -- | -- | -- | -- | -- | **91,5** | -- | 115,5 | -- | -- | -- |
| Glycerol | 85,5 | -- | -- | -- | -- | -- | 74,5 | **148,5** | 112,5 | -- | -- | -- |

Supplementary Table 4: Overview of fluorescence penetration and SNR in depth in complex 3D cell cultures. Melanoma triculture spheroids and Dynarray chip-based co-cultures of MDA-MB-231-ECFP and CCD-Sk1137 cells were fixed, stained with DAPI (Melanoma triculture) or DRAQ5 (Dynarray culture), followed by optical tissue clearing or embedding as indicated and subsequent confocal whole mount microscopy. In tricultures, melanoma cells were labeled prior to seeding with CellTracker Green, in Dynarray cultures, CCD-Sk1137 cells were marked with CellTracker Red. The table reports the average values for absolute and normalized depth of 50 % signal loss, 90 % signal loss, and SNR < 5 for DAPI and Cell Tracker Green (tri-culture) or CellTracker Red and DRAQ5 (Dynarrays) in the absence of z-compensation. N ≥ 5. For clarity, maximal values are depicted with green shading.
